# Synthesis and Biological Evaluation of 2-Methyl-4,5-Disubstituted Oxazoles as a Novel Class of Highly Potent Antitubulin Agents

**DOI:** 10.1038/srep46356

**Published:** 2017-04-13

**Authors:** Romeo Romagnoli, Pier Giovanni Baraldi, Filippo Prencipe, Paola Oliva, Stefania Baraldi, Maria Kimatrai Salvador, Luisa Carlota Lopez-Cara, Andrea Brancale, Salvatore Ferla, Ernest Hamel, Roberto Ronca, Roberta Bortolozzi, Elena Mariotto, Elena Porcù, Giuseppe Basso, Giampietro Viola

**Affiliations:** 1 Dipartimento di Scienze Chimiche e Farmaceutiche, Università di Ferrara, 44121 Ferrara, Italy; 2 Departamento de Química Farmacéutica y Orgánica, Facultad de Farmacia, Campus de Cartuja s/n, 18071, Granada, Spain; 3 School of Pharmacy and Pharmaceutical Sciences, Cardiff University, King Edward VII Avenue, Cardiff, CF10 3NB, UK; 4 Screening Technologies Branch, Developmental Therapeutics Program, Division of Cancer Treatment and Diagnosis, Frederick National Laboratory for Cancer Research, National Cancer Institute, National Institutes of Health, Frederick, Maryland 21702, USA; 5 Dipartimento di Medicina Molecolare e Traslazionale Unità di Oncologia Sperimentale ed Immunologia, Università di Brescia, 25123 Brescia, Italy; 6 Dipartimento di Salute della Donna e del Bambino, Laboratorio di Oncoematologia, Università di Padova, 35131 Padova, Italy

## Abstract

Antimitotic agents that interfere with microtubule formation are one of the major classes of cytotoxic drugs for cancer treatment. Multiple 2-methyl-4-(3′,4′,5′-trimethoxyphenyl)-5-substituted oxazoles and their related 4-substituted-5-(3′,4′,5′-trimethoxyphenyl) regioisomeric derivatives designed as *cis*-constrained combretastatin A-4 (CA-4) analogues were synthesized and evaluated for their antiproliferative activity *in vitro* against a panel of cancer cell lines and, for selected highly active compounds, interaction with tubulin, cell cycle effects and *in vivo* potency. Both these series of compounds were characterized by the presence of a common 3′,4′,5′-trimethoxyphenyl ring at either the C-4 or C-5 position of the 2-methyloxazole ring. Compounds **4g** and **4i**, bearing a *m*-fluoro-*p*-methoxyphenyl or *p*-ethoxyphenyl moiety at the 5-position of 2-methyloxazole nucleus, respectively, exhibited the greatest antiproliferative activity, with IC_50_ values of 0.35-4.6 nM (**4g**) and 0.5–20.2 nM (**4i**), which are similar to those obtained with CA-4. These compounds bound to the colchicine site of tubulin and inhibited tubulin polymerization at submicromolar concentrations. Furthermore, **4i** strongly induced apoptosis that follows the mitochondrial pathway. *In vivo*, **4i** in a mouse syngeneic model demonstrated high antitumor activity which significantly reduced the tumor mass at doses ten times lower than that required for CA-4P, suggesting that **4i** warrants further evaluation as a potential anticancer drug.

Cellular microtubules, undergoing constant assembly and disassembly from α,β-tubulin heterodimers, are key components of the cytoskeleton and are involved in a wide range of cellular functions. Perhaps their most important role is formation of the mitotic spindle to direct cell division and proper chromosomal separation^1^. Antimitotic agents represent a major class of cytotoxic drugs for cancer treatment, and tubulin is the target for numerous small natural and synthetic molecules that inhibit the formation of the mitotic spindle^2^,^3^,^4^. Among the naturally occurring antimicrotubule agents, one of the most active is the *cis-*stilbene combretastatin A-4 (CA-4, **1a**, Fig. 1), isolated from the African cape bushwillow *Combretum caffrum*^5^. CA-4 inhibits tubulin assembly by interacting with β-tubulin at the colchicine site^6^. The corresponding water soluble prodrug salt, CA-4 disodium phosphate (CA-4P, **1b**)^7^, is currently in Phase II trials for anaplastic thyroid cancer^8^, and it was found to have potent activity in reducing tumor blood flow, thus acting as a vascular disrupting agent (VDA)^9^. Its structural simplicity, along with its ability to selectively damage tumor vasculature, makes CA-4 of great interest from the medicinal chemistry point of view^10^.

Previous SAR studies have demonstrated that both the 3′,4′,5′-trimethoxy substitution pattern on the A-ring and the *cis*-olefin configuration at the bridge were fundamental requirements for optimal activity, while some B-ring structural modifications were tolerated by the target^11^. Numerous researchers have undertaken modification of the relatively unstable *cis*-double bond of CA-4, which is characterized by the tendency to undergo isomerization to the biologically inactive *trans*-form in solution during storage, administration and metabolism^12^. Thus, to retain the appropriate geometry of the two adjacent aryl groups required for potent bioactivity, chemically stable *cis*-restricted derivatives of CA-4 with general structure **2** were obtained by incorporation of the stilbene double bond into vicinally diaryl-substituted five-member aromatic heterocyclic rings, such as pyrazole^13^, imidazole^13^,^14^, thiazole^15^, furazan (1,2,5-oxadiazole)^16^, isoxazole^17^, oxazole^13^,^14^, 1,2,3-thiadiazole^18^, triazole^19^ and 1,2,3,4-tetrazole^20^.

Among our efforts focused on modification of the *cis*-double bond of CA-4, we previously described a series of 2-methyl-4-(3′,4′,5′-trimethoxyphenyl-5-substituted thiazoles with general structure **3** that showed moderate antiproliferative activity against a panel of five cancer cell lines. These compounds were one to three orders of magnitude less active than CA-4, in terms of molar IC_50_ values^21^. These compounds also caused accumulation of HeLa and Jurkat cells in the G2/M phase of the cell cycle, as is typical for antimicrotubule agents. Among the synthesized compounds, derivative **3d** (R_1_ = naphth-2-yl) was the most active as an inhibitor of tumor cell growth, with IC_50_ values ranging from 33 to 702 nM in the five cell lines examined but was comparable to CA-4 as an inhibitor of tubulin polymerization.

In our ongoing effort to discover novel and potent antimicrotubule agents, these results led us to start a pharmacophore exploration and optimization effort around the 2-methylthiazole derivatives with general formula **3**. Here we describe replacing the thiazole nucleus with the less aromatic and basic bioisosteric equivalent oxazole ring^22^, by the preparation of two different regioisomeric series of 2-methyl-4,5-disubstituted oxazole derivatives with general structures **4** and **5**. In these two series of designed analogues, obtained by interchanging the substitution pattern of ring A and B, we fixed one of the aryl groups as the 3′,4′,5′-trimethoxyphenyl moiety, identical to the A-ring of CA-4, and examined several substitutions with electron-withdrawing (F and Cl) or electron-releasing (Me, OMe, and OEt) groups (EWG or ERG, respectively) on the other aryl moiety, corresponding to the B-ring of CA-4. In addition, for compounds **4a** and **5a**, the B-ring of CA-4 was replaced with the bulky and lipophilic naphth-2-yl moiety.

It has been previously reported that the replacement of the *meta*-hydroxy group of ring B of CA-4 with halogens such as fluorine or chlorine increased tubulin affinity as well as antiproliferative potency^23^. Since the methoxy and ethoxy groups proved to be favorable for bioactivity, we maintained one of these substituents at the *para*-position and introduced an additional substituent (F and Cl) at the *meta*-position of the phenyl ring.

## Chemistry

**Figure 2:**
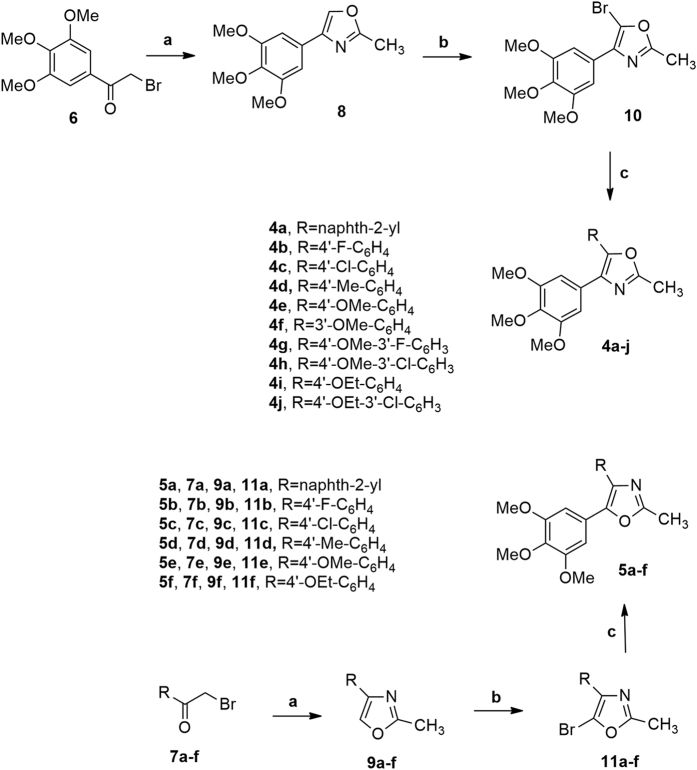
Reagents. a: CH_3_CONH_2_, 150 °C, 2 h; b: *N*-bromosuccinimide, CHCl_3_, rt; c: PdCl_2_(DPPF), ArB(OH)_2_, CsF, 1,4-dioxane. 65 °C.

Synthesis of compounds **4a-j** and **5a-f** was accomplished using a three-step procedure described in Fig. 2. 2-Methyl-4-substituted oxazole derivatives **8** or **9a-f** were prepared by the condensation of 2-bromo-1-(3′,4′,5′-trimethoxyphenyl)ethanone **6** and the appropriate α-bromo acetophenone **7a-f**, respectively, with acetamide at 150 °C for 2 h. The subsequent chemoselective monobromination at the 5-position of derivatives **8** and **9a-f** with *N*-bromosuccinimide in CHCl_3_ furnished the 2-methyl-4-substituted-5-bromooxazole analogues **10** or **11a-f**, respectively. Finally, these latter intermediates were subjected to Suzuki cross-coupling reaction with the appropriate arylboronic acid under heterogeneous conditions [PdCl_2_(DPPF), CsF] in 1,4-dioxane at 65 °C, to furnish the target 2-methyl-4-(3′,4′,5′-trimethoxyphenyl)-5-substituted and isomeric 2-methyl-4-substituted-5-(3′,4′,5′-trimethoxyphenyl)oxazole derivatives **4a-j** and **5a-f**, respectively.

## Biological Results and Discussion

### *In vitro* antiproliferative activities

The 2-methyl-4-(3′,4′,5′-trimethoxyphenyl)-5-substituted oxazoles **4a-j** and the corresponding isomeric 2-methyl-4-substituted-5-(3′,4′,5′-trimethoxyphenyl) oxazole analogues **5a-f** were evaluated for their antiproliferative activity against a panel of seven human tumor cell lines in comparison with the reference compounds CA-4 and 2-methyl-4-(4′-methoxyphenyl)-5-(3′,4’,5′-trimethoxyphenyl)thiazole **3e** (Table 1). Two of the synthesized compounds, **4g** and **4i**, had the best antiproliferative activities against these cell lines and, overall, were significantly more active than the rest of derivatives as well as than CA-4. Specifically, the *m*-fluoro-*p*-methoxyphenyl derivative **4g** and the *p*-ethoxyphenyl analogue **4i** exhibited IC_50_ values of 0.35–4.6 nM and 0.5–20 nM, respectively, as compared with the range 0.8–3100 nM obtained with CA-4. Derivative **4g** was equipotent to CA-4 against Jurkat and SEM cells, while it was from 2- to 940-fold more active against the other five cancer cell lines. Compound **4i** was 2-fold less active than CA-4 against RS4;11 cells, equipotent to CA-4 against Jurkat and SEM cells but 4- to 153-fold more potent against the other four cell lines. Compounds **4a**, **4e**, **4j** and **5f** also inhibited the growth of most of the cancer cell lines at single- to low double-digit nanomolar concentrations.

The relative positions of the two aromatic rings on the 2-methyloxazole core seemed to be critical for antiproliferative activity. In examining the effect of switching the position of the two aromatic rings at the 4- and 5-positions on the 2-methyloxazole system (**4a** *vs.* **5a**, **4b** *vs.* **5b**, **4c** *vs.* **5c**, **4d** *vs.* **5d**, **4e** *vs.* **5e**, **4i** *vs.* **5f**), we observed a considerable difference in potency between the 4′-(3′,4′,5’-trimethoxyphenyl) derivatives **4a-e** and **4i** and the regioisomeric 5-(3′,4′,5′-trimethoxyphenyl)oxazole counterparts **5a-f**. Generally, the latter compounds were less active than the former against all the cancer cell lines. Moreover, comparing **3e** and **5e**, which shared common 4′-methoxyphenyl and 3′,4′,5′-trimethoxyphenyl moieties at their 4 and 5-positions, the thiazole derivative **3e** was from 300- to 11-fold less active than its oxazole congener **5e**.

The 5-(2′-naphthyl)oxazole derivative **4a** was more active than CA-4 in four of the seven cancer cell lines, with activity from single to double digit nanomolar concentrations (IC_50_: 0.5–73.2 nM). The isomer derivative **5a** was from 4- to 630-fold less active than **4a**, with the greatest reduction of activity against the HT-29 cells.

In comparing the effect of EWG’s or ERG’s at the *para*-position of the phenyl ring, we observed that compounds with electron-withdrawing substituents such as F (**4b** and **5b**) and Cl (**4c** and **5c)** showed reduced antiproliferative activity compared to their counterparts with electron-releasing Me, OMe or OEt moieties (**4d-e**, **4i** and **5d-f**).

The *p*-fluorophenyl derivative **4b** and its regioisomer **5b** were the least active compounds of the series, with IC_50_ values over 9 μM against all cell lines screened. Increasing the size of the halide from fluorine to chlorine lead to an increase of activity with all seven cell lines, which was most pronounced against the RS4;11 cell line. Replacing the halogen with the small electron-releasing methyl group at the *p*-position of the phenyl group (**4d**) improved significantly the antiproliferative activity relative to **4b** and **4c**, with IC_50_ values ranging from 25 to 243 nM, with double digit nanomolar activity against HeLa, A549, HT-29 and RS4;11 cells. The *p*-tolyl derivative **4d** was from 3- to 28-fold more potent than isomeric counterpart **5d**, and this difference was most pronounced against A549 and RS4;11.

Replacement of the methyl group with the stronger electron-releasing methoxy group (compounds **4d** and **4e**, respectively) increased the activity from 3- to 9-fold on five of the seven cancer cell lines. For the two 4-(3′,4′,5′-trimethoxyphenyl)oxazole analogues **4e** and **4f**, the position of methoxy substituent on the 5-phenyl ring had a profound influence on antiproliferative activity. Moving the methoxy group from the *para*- (**4e**) to the *meta*-position (**4f)**, led to a drastic reduction in antiproliferative activity. Compound **4e** was 2- to 3-fold more active than the regioisomeric derivative **5e**, except in HT-29 cells, where **5e** was 3-fold more active than **4e**.

Relative to the activity of **4e**, the insertion of an additional EWG on the *meta*-position of the *p*-methoxyphenyl ring had varying effects on antiproliferative activity. The marked influence of an additional fluorine at the *meta*-position of **4e**, to furnish the *m*-F-*p*-OMe derivative **4g**, led to a 4–276-fold increase in antiproliferative activity, which was most pronounced in the A549 cells. An opposite effect occurred with replacement of *m*-fluorine with *m*-chlorine, to furnish derivative **4h**, which led to a 60–213-fold reduction of activity relative to **4g**. Compound **4h** was also 5–60-fold less active than **4e** against six of the seven cancer cell lines, the exception being the A549 cells.

The *p*-ethoxyphenyl homologue **4i** was 2- to 358-fold more potent than its methoxy counterpart **4e**. The greatest difference in activity was 358-fold against the A549 cells. Since the *p*-ethoxy group of **4i** was favorable for potency, the introduction of an additional electron-withdrawing chlorine group at the *meta*-position of the *p*-ethoxyphenyl ring, resulting in compound **4j**, had variable effects, producing a 5–40-fold reduction in antiproliferative activity against five of the cell lines and increased activity against MCF-7 and HT-29 cells. Compound **4i** was from 2- to 12-fold more potent than the isomeric derivative **5f** in five of the cell lines, the exceptions being the HT-29 and MCF-7 cells, in which **5f** was 11- and 3-fold more active than **4i**, respectively.

### Effects of test compounds 4a, 4i and 5f in non tumoral cells

To obtain a preliminary indication of the cytotoxic potential of these derivatives in normal human cells, some of the most active compounds (**4a**, **4i** and **5f**) were evaluated *in vitro* against peripheral blood lymphocytes (PBL) from healthy donors. All compounds showed an IC_50_ greater than 10 μM both in quiescent lymphocytes and in lymphocytes in an active phase of proliferation induced by phytohematoagglutinin (PHA) a mitogenic stimulus (Table 2). Moreover, we also evaluated the effects of these compounds on primary cultures of human umbilical endothelial cells (HUVECs), and we found that the three compounds were practically inactive, having IC_50_ values >100 μM. These results indicate that these compounds have very low toxicity in normal cells in comparison to tumor cells, suggesting potential for an excellent therapeutic index.

### Inhibition of tubulin polymerization and colchicine binding

A subset of compounds (**4a, 4d**,**e, 4g**, **4i**,**j**, **5a**, **5e**,**f**) were evaluated for their *in vitro* inhibition of tubulin polymerization and for inhibitory effects on the binding of [^3^H]colchicine to tubulin (Table 3). CA-4 was also examined in contemporaneous experiments. In the assembly assay, with 10 μM tubulin, one of the most active antiproliferative agents (**4i**), along with compound **4d**, were the best inhibitors of tubulin polymerization, with IC_50_ values of 0.56 and 0.66 μM, respectively, having twice the potency of CA-4 (IC_50_:1.3 μM). Derivatives **4a**, **4g** and **5f** showed comparable antitubulin activity to that of CA-4, while compound **5e** was about half as potent as CA-4. For these new compounds and CA-4, the order of inhibitory effects on tubulin assembly was **4i** > **4d** > **4e** > **4g** = **4a** = **5f** = CA-4 > **5a** > **4j** > **5e**.

In the colchicine binding studies, the same compounds potently inhibited the binding of [^3^H]colchicine to tubulin, since 54–86% inhibition occurred with these agents and colchicine both at 5 μM. Specifically, derivatives **4d** and **4i** were slightly less active than CA-4 (86 and 82% inhibition, respectively), which in this experiment inhibited colchicine binding by 99%. Inhibition of colchicine binding by compounds **4j**, **5a** and **5e** fell into the 53–64% range.

While this group of compounds were all highly potent in the biological assays (inhibition of cell growth, tubulin assembly and colchicine binding), correlations between these assay types were imperfect. Thus, while compound **4g** was half as active as an assembly inhibitor as **4d**, these two compounds were equipotent in the colchicine binding assay. Moreover, compounds **4d** and **4i** were nearly equipotent as inhibitors of tubulin assembly, while **4i** was 2-185-fold more active than **4d** in its effects on cell growth.

Nevertheless, these studies identified tubulin as the molecular target of these compounds, since those with the greatest inhibitory effects on cell growth strongly inhibited tubulin assembly and the binding of colchicine to tubulin.

### Molecular modelling

**Figure 3:**
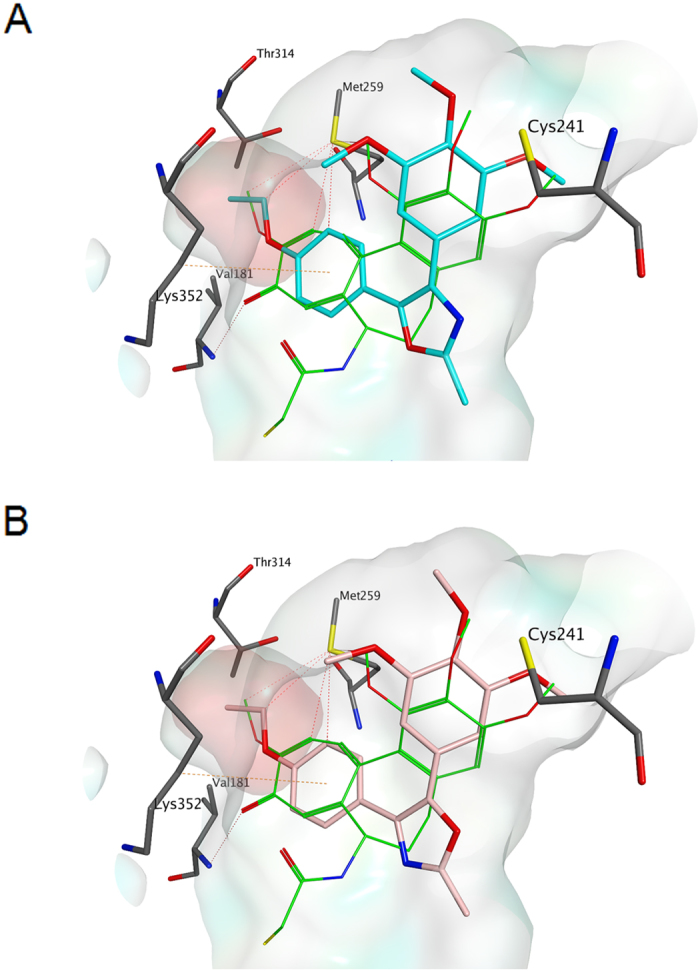
Proposed binding modes for compounds 4i (carbon atoms in magenta, Fig. 1A) and 5f (carbon atoms in pink, Fig. 1B) in the colchicine site. Co-crystallized DAMA-colchicine is shown with carbon atoms in green. The hydrophobic subpocket referred to in the text is highlighted with a pink surface.

The binding mode in the colchicine site of tubulin of the newly prepared 2-methyloxazole derivatives was elucidated performing a series of molecular docking simulations, following a previous reported procedure^21^. The binding observed for all the derivatives is closely related to the one found for the co-crystallized DAMA-colchicine, and it is consistent with those previously reported for different tubulin polymerization inhibitors^21^,^23^. The trimethoxyphenyl ring, in both the 4-(*para*-ethoxyphenyl) and isomeric 5-(*para*-ethoxyphenyl)-2-methyloxazole derivatives **4i** and **5f**, respectively, is in close contact with Cys241, while the second substituted phenyl ring occupies a small hydrophobic subpocket (Fig. 3, Panels A and B), with the ethoxy substituents lying deep in this pocket. Hydrophobic interactions with the surrounding amino acids (e.g., βMet259, βThr314, βVal181, etc.) of the subpocket stabilize the binding of the molecules in the colchicine site.

### Analysis of cell cycle effects

**Figure 4:**
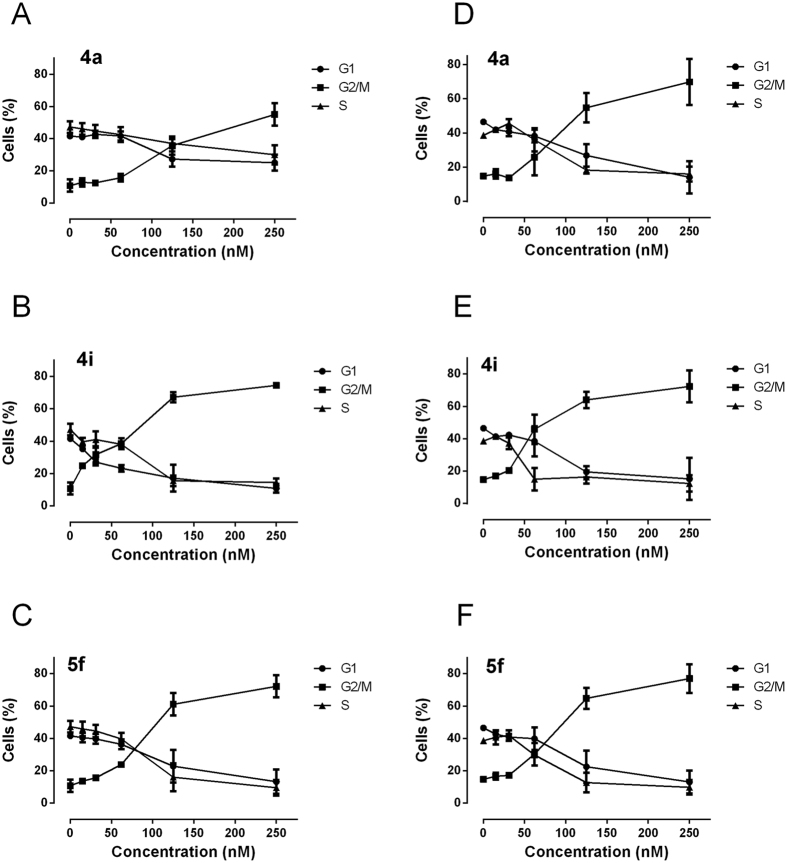
Percentage of cells in each phase of the cell cycle in Jurkat (Panels A–C) and HeLa cells (Panels D–F) treated with the indicated compounds at the indicated concentrations for 24 h. Cells were fixed and labeled with PI and analyzed by flow cytometry as described in the experimental section. Data are shown as mean ± SEM of two independent experiments.

**Figure 5:**
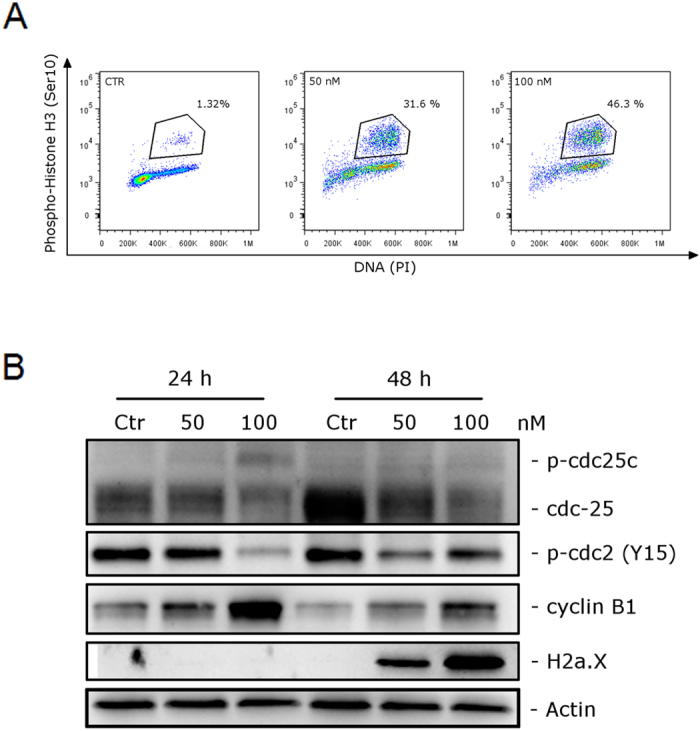
(**A**) Representative histograms of mitotic cells with phosphorylated histone-H3 after treatment with **4i** at the indicated concentrations in HeLa cells. Data are representative of two experiments with similar results. (**B**) Effect of **4i** on cell cycle checkpoint proteins and expression of p-H2A.X^Ser139^. HeLa cells were treated for 24 or 48 h with the indicated concentrations of **4i**. The cells were harvested and lysed for detection of the expression of the indicated protein by western blot analysis. To confirm equal protein loading, each membrane was stripped and reprobed with anti-β-actin antibody.

The effects of a 24 h treatment with different concentrations of **4a**, **4i** and **5f** on cell cycle progression in Jurkat, and HeLa cells were determined by flow cytometry (Fig. 4, Panels A–F). All three compounds caused a significant G2/M arrest in a concentration-dependent manner in the two cell lines examined, with a rise in G2/M cells occurring at a concentration as low as 50 nM, especially with compound **4i**, while at higher concentrations more than 60% of the cells were arrested in G2/M. The cell cycle arrest in G2/M phase was accompanied by a corresponding reduction in cells in the other phases (G1 and S) of the cell cycle. This ability of **4i** to induce G2/M arrest correlates directly with its strong inhibition of tubulin polymerization. With the purpose of evaluating whether **4i** arrested cells in mitosis, Hela cells were stained with an immunofluorescent antibody to p-histone H3, a well known mitotic marker^24^, as well as with propidium iodide (PI), and analyzed by flow cytometry. As shown in Fig. 5 (Panel A), in which representative histograms are presented, cells arrested in M phase by treatment with **4i** are readily distinguished from G2 cells by the higher level of p-histone H3. Compound **4i** induced, after a 24 h incubation, a dose-dependent increase in the percentage of mitotic cells, from 1.3% observed in the untreated cells to about 32% and 46% at 50 and 100 nM **4i**, respectively.

### Compound 4i induced alteration of cell cycle checkpoint proteins and induced DNA damage

We investigated the effects of **4i** on the expression of proteins involved in regulation of the cell cycle and in spindle assembly. Cyclin B1 is involved in the G2 to M transition as a complex with cdc2, and the activation of the cdc2/cyclin B1 complex through cdc25c-dependent dephosphorylation of phospho-cdc2 and phosphorylation of cyclin B1 triggers cells to enter mitosis^25^,^26^. As shown in Fig. 5 (Panel B) a marked increase of cyclin B1 occurred in a concentration dependent manner following 24 and 48 h treatments with **4i**. On the other hand, total cdc25c expression was reduced both at 24 and 48 h after treatment with 100 nM **4i**, and we observed the simultaneous appearance of a slowly migrating form of cdc25c, indicating changes in its phosphorylation state. Furthermore, in good agreement, the expression of phosphorylated cdc2 decreased, most noticeable after the 24 h treatment with 100 nM **4i**. These findings are in good agreement with previous results^21^,^23^ obtained with other antimitotic derivatives and indicate that cdc2/cyclin B1 complexes failed to be activated, preventing cells from exiting mitosis, which would eventually lead to apoptotic cell death.

Moreover, since it is well known that prolonged mitotic arrest induces DNA damage^27^,^28^ we also examined the expression of phosphorylated histone H2A.X at Ser139 (γH2A.X), a marker of DNA damage^29^. We observed (Fig. 5, Panel B) a great increase of the phosphorylation of γH2A.X, after a 48 h treatment, suggesting that DNA damage occurred following treatment with **4i**.

### Compound 4i induced apoptosis

**Figure 6:**
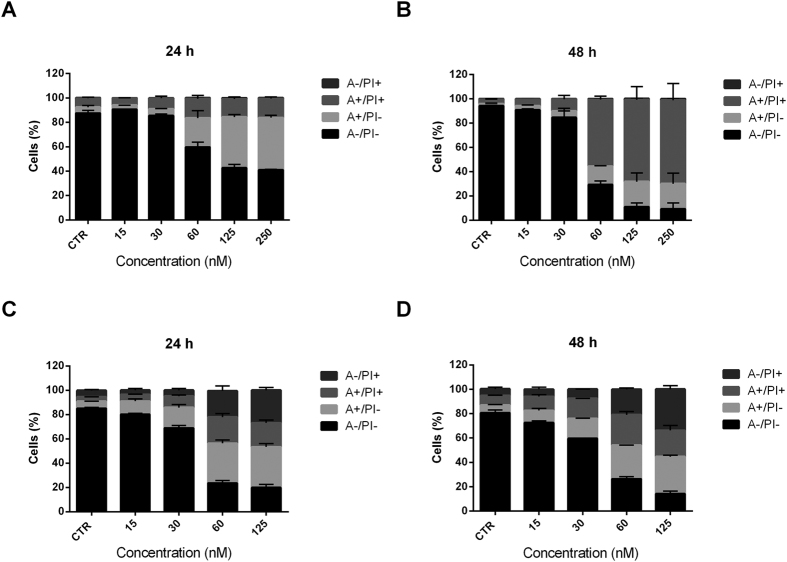
Flow cytometric analysis of apoptotic cells after treatment of Jurkat cells (Panels A and B) or HeLa cells (Panels C and D) with **4i** at the indicated concentrations after incubation for 24 or 48 h. The cells were harvested and labeled with annexin-V-FITC and PI and analyzed by flow cytometry. Data are represented as mean ± SEM of three independent experiments.

To evaluate the mode of cell death induced by **4i**, we used an annexin-V/PI assay. We treated two cell lines (HeLa and Jurkat) with the test compound with concentrations ranging from 15 to 125 nM for 24 or 48 h. As shown in Fig. 6, both Jurkat (Panels A, B) and HeLa (Panels C, D) cells treated with **4i** showed a significant accumulation of annexin-V positive cells in a concentration dependent manner after a 24 h treatment, and the proportions of apoptotic cells further increased at 48 h. Note that **4i** caused the appearance of 70% apoptotic cells at 60 nM in Hela cells, while in the leukemic cell line we observed a lower value (40%) after a 24 h treatment, in good agreement with its cytotoxic activity.

### Compound 4i induced apoptosis through the mitochondrial pathway

**Figure 7:**
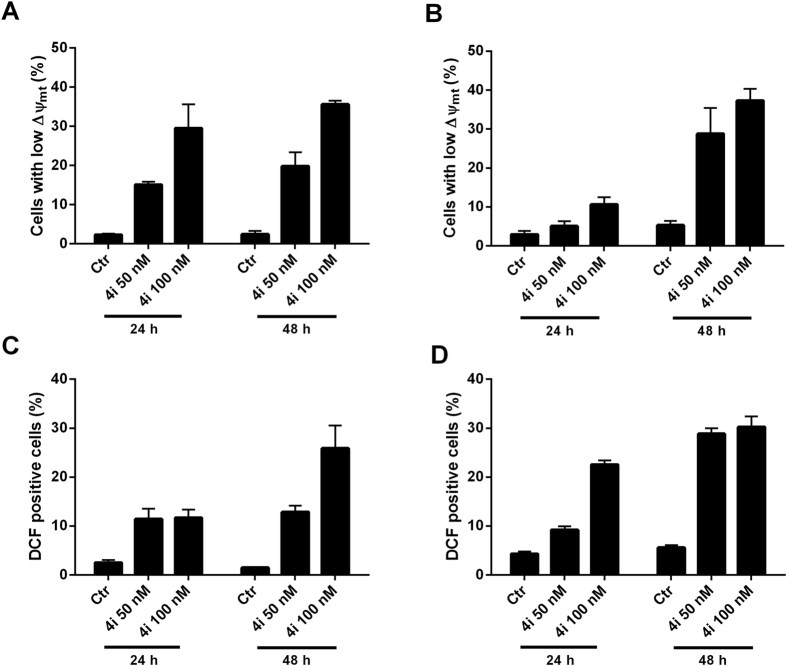
Assessment of mitochondrial membrane potential (Panels A and B) and ROS production after treatment of Jurkat cells (A and C) or HeLa cells (B and D) with compound **4i**. Cells were treated with the indicated concentration of compound for 24 or 48 h and then stained with the fluorescent probes JC-1 or H_2_-DCFDA and analyzed by flow cytometry. Data are presented as mean ± S.E.M. for three independent experiments.

Since many combretastatin analogues cause apoptosis following the mitochondrial pathway [19c, refs 20 and 23] we investigated if **4i** also induced mitochondrial depolarization. Mitochondrial potential was monitored by flow cytometry using the fluorescent dye JC-1. We treated both Hela and Jurkat cells with **4i** at 50 or 100 nM for 24 or 48 h. As shown in Fig. 7 (Panels A and B), both Jurkat and HeLa cells treated with **4i** exhibited a marked increase in the percentage of cells with low Δψ_mt_ in a time dependent manner, paralleling the results obtained with the annexin-V apoptotic assay. Since it is well known that dissipation of mitochondrial potential is associated with mitochondrial production of reactive oxygen species (ROS)^30^,^31^, we also evaluated whether ROS production increased after treatment with **4i**. We utilized the dye 2,7-dichlorodihydrofluorescein diacetate (H_2_-DCFDA), which is oxidized to the fluorescent compound dichlorofluorescein (DCF) upon ROS induction.

The results shown in Fig. 7 (Panels C and D) indicate that **4i** induced ROS production in comparison with the amounts observed in control cells, in both Jurkat and HeLa cells, moving from about 4% of DCF positive cells in untreated samples to about 25–30% in treated cells at 100 nM, the highest concentration examined. These results are in excellent agreement with the dissipation of Δψ_mt_ described above.

### Compound 4i induced PARP cleavage and down regulation of anti-apoptotic proteins

**Figure 8:**
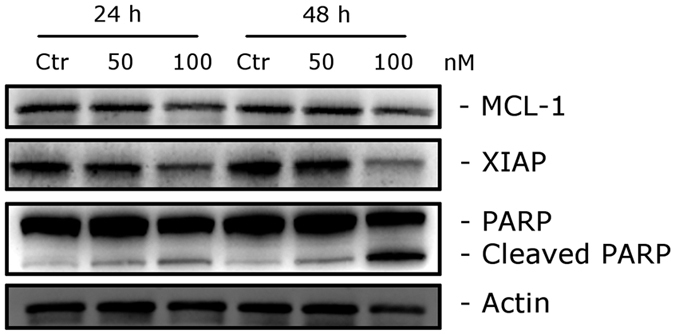
Western blot analysis of caspase-3, PARP, Mcl-1 and XIAP after treatment of HeLa cells with **4i** at the indicated concentrations and for the indicated times. To confirm equal protein loading, each membrane was stripped and reprobed with anti-β-actin antibody.

To further study the apoptotic process induced by **4i**, we analyzed the cleavage of PARP a typical marker of apoptosis^32^. As shown in Fig. 8, immunoblot analysis of Hela cells treated with 50 or 100 nM **4i** indicated the activation PARP after both 24 and 48 h, as evidenced by the appearance of its cleavage fragments.

We also investigated the expression of two anti-apoptotic proteins, Mcl-1 and XIAP. Mcl-1 is a member of the Bcl-2 family of anti-apoptotic proteins. Mcl-1 is overexpressed in many cancers, and it has been reported that sensitivity to antimitotic drugs is regulated by Mcl-1 levels^33^. As shown in Fig. 8, the expression of Mcl-1 was only slightly decreased at the highest **4i** concentration used (100 nM).

On the other hand, XIAP, also a member of the IAP family (inhibitors of apoptosis protein)^34^, was significantly reduced after both the 24 and 48 h treatments, suggesting that **4i** treatment induced downregulation of these proteins to disable their anti-apoptotic function.

### Evaluation of antivascular activity of 4i and 5f

**Figure 9:**
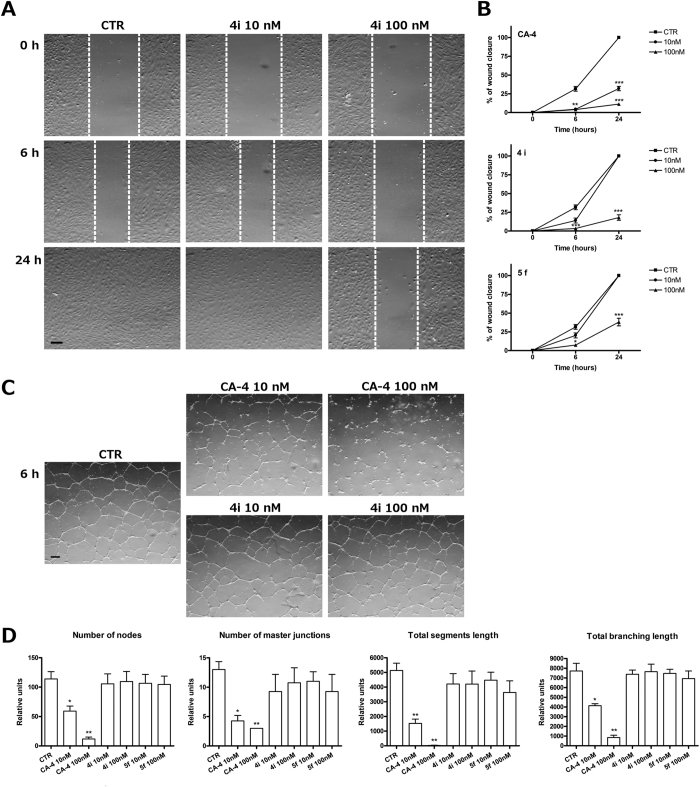
Antivascular activity of compounds **4i** and **5f**. (**A**) Representative images of confluent HUVEC monolayers that were scratch wounded and treated with vehicle (Ctr) or 10 or 100 nM **4i**, as indicated, after 0, 6 or 24 h from the scratch. After different times, cells were photographed (10x magnification), and the scratch width was quantified (Panel B). **CA-4** was chosen as reference compound. Data are presented as the mean ± SEM of three independent experiments. ***p < 0.01 vs control. (**C**) Disruption of capillary-like structures. Representative images of HUVEC cells that were incubated on Matrigel until the formation of capillary-like tubes. The disrupting effects of 10 or 100 nM **4i** are shown (10x magnification). D. Quantification of the effect of compounds **4i**, **5f** and **CA-4** on dimensional and topological parameters of HUVEC morphology in comparison to time zero. Data are presented as the mean ± SEM of three independent experiments. *p < 0.01 vs Ctr.

Since many tubulin binding agents, including CA-4, are endowed with vascular disrupting activity [9c], we investigated *in vitro* the potential anti-vascular activity of compounds **4i** and **5f**. We tested the anti-vascular effects in HUVECs, evaluating the ability of the compounds to i) interfere with angiogenesis by inhibiting endothelial cell migration and ii) to interfere with the process of capillary-like tube formation. Confluent HUVEC monolayers were scraped with a pipette tip and cellular migration induced to repair the wound was followed by optical microscopy, and the percentage of reduction of wound healing was calculated at different times^35^. As shown in Fig. 9 (Panels A and B), after only a 6 h treatment, cell migration was significantly reduced at 100 nM compound **4i** or **5f**. This significant reduction was maintained for a 24 h treatment as well. In contrast, CA-4 was significantly active even at 10 nM, after both the 6 and 24 h treatments.

Endothelial cells seeded on Matrigel are able to form a capillary network miming the first angiogenesis steps^36^. This network is a useful experimental model to assess the action of molecules on vascular morphogenesis.

The antivascular effect induced by the tested compounds is shown in Fig. 9 (Panels C and D). In this case, we evaluated the pictures taken after a 24 h incubation by optical microscopy (Fig. 9, Panel C), and a quantitative analysis was carried out studying two dimensional (percent area covered by HUVECs and total length of HUVECs network per field) and two topological parameters (number of meshes and branching points per field).

Compounds **4i** and **5f** did not show a significant antivascular effect on Matrigel preformed tubular structures (Fig. 9, Panel D), even at the highest concentration used (100 nM). In this assay, CA-4 showed, as expected, a significant antivascular activity even at 10 nM. Our findings suggest that these compounds do not possess a good antivascular profile in comparison to the well known activity of CA-4, since some effects, in particular cellular migration, occurred at a concentration higher than that required for CA-4.

### Compound 4i induced tumor growth reduction in a mouse allograft tumor model

**Figure 10:**
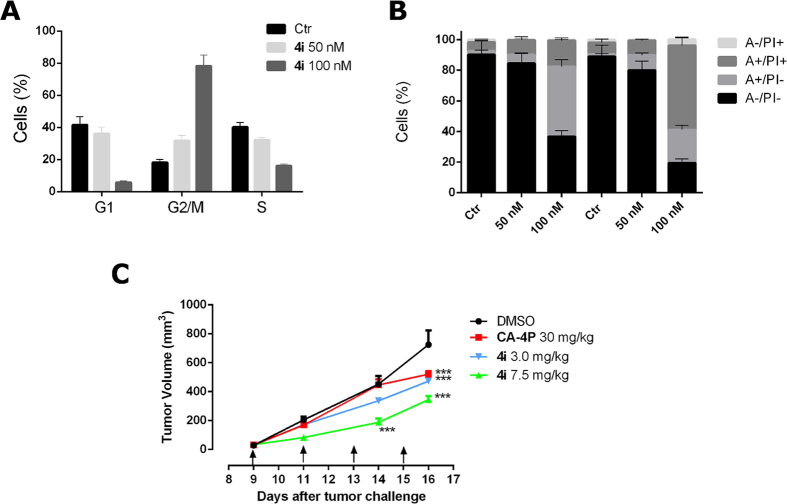
Panel A. Effect of compound **4i** on cell cycle of BL6-B16 murine melanoma cells treated with the compounds at the indicated concentrations for 24 h. Cells were fixed and labeled with PI and analyzed by flow cytometry as described in the experimental section. Data are shown as mean ± SEM of two independent experiments. Panel B. Flow cytometric analysis of apoptotic cells after treatment of BL6-B16 murine melanoma with **4i** at the indicated concentrations after incubation for 24 or 48 h. The cells were harvested and labeled with annexin-V-FITC and PI and analyzed by flow cytometry. Data are represented as mean ± SEM of two independent experiments. Panel C. Inhibition of mouse allograft growth *in vivo* by compound **4i**. Male C57BL/6 mice were injected subcutaneously at their dorsal region with 10^7^ BL6-B16 murine melanoma cells. Tumor-bearing mice were administered the vehicle, as control, or 3 or 7.5 mg/kg of **4i** or CA-4P as reference compound at the dose of 30 mg/kg. Injections were given intraperitoneally at the days indicated by the arrows. Data are presented as mean ± SEM of tumor volume at each time point for 5 animals per group. ***p < 0.001 *vs*. control.

The antitumor effect *in vivo* of compound **4i** was evaluated in an allograft tumor model developed in mice^37^. This model consists of the use of B16 murine melanoma cells that are injected in the flank of mice. Consequently, in preliminary experiments we wanted to verify the effectiveness of the compound **4i** on this murine tumor line. The compound had an IC_50_ of 15.6 ± 1.9 nM measured by the MTT assay, indicating that its cytotoxic potency was similar to that found in human tumor cell lines (see Table 1). In addition, we also evaluated, if in this cell line, the compound was able to arrest cells in the G2/M phase of the cell cycle and to induce apoptosis. As shown in Fig. 10 (Panels A and B), **4i**, as was observed in the human tumor cell lines, induced a G2/M arrest and a strong apoptotic response at low concentrations (50–100 nM). These results indicated that the allogeneic mouse model used was fully relevant to the evaluation of **4i** *in vivo*. Thus, **4i** was administered by the intraperitoneal route every other day, at two different doses (3.0 and 7.5 mg/kg). As reference compound, CA-4P (**1b**) was used at 30 mg/kg.

As shown in Fig. 10 (panel C), after a six day treatment (doses administered on days 9, 11 and 14), **4i** was able to significantly reduce tumor burden in a dose-dependent manner, even at the lowest dose tested (3.0 mg/kg). We observed reduction in tumor mass of 34.9, and 52.5% at the doses of 3.0 and 7.5 mg/kg, respectively. The reference compound CA-4P at 30 mg/kg induced only a 28.0% reduction in tumor mass. Notably, the *in vivo* efficacy clearly indicate an increased antitumor efficacy of **4i** as compared with CA-4P (in both weight and molar terms). Even at the highest dose, **4i** did not show any sign of toxicity and did not cause a decrease in animal body weight (data not shown).

## Conclusions

The isomerization of the *cis*-double bond of CA-4 to its t*rans*-form in solution is one of the major disadvantages of this molecule. The instability of the *Z*-double bond of CA-4 has been resolved by incorporating the stilbene double bond into the structure of five-member heterocyclic rings. The bioisosteric equivalence between oxazole and thiazole prompted us to synthesize by a three-step procedure two novel series of 2-methyl-4,5-disubstituted oxazole derivatives with general formulas **4** and **5**, in which the oxazole ring replaced the thiazole system of previously published analogues with general structure **3** and could serve as a suitable mimic to retain the bioactive configuration afforded by the *cis*-double bond present in CA-4. For both these series of compounds, the 3′,4′,5′-trimethoxyphenyl and 2-methyloxazole rings mimic the ring A and *cis*-double bond of CA-4, respectively, while a naphth-2-yl or phenyl ring substituted with electron-releasing or electron-withdrawing groups was utilized as a B-ring surrogate to mimic the 3′-hydroxy-4′-methoxyphenyl group in CA-4. Comparing compounds with the same aryl substitution, the 2-methyl-4-(3′,4′,5′-trimethoxyphenyl)oxazole derivatives were more active than their isomeric 2-methyl-5-(3′,4′,5′-trimethoxyphenyl)oxazole counterparts. The results indicated that 2-methyloxazole derivatives **4a**, **4d**-**e**, **4i** and **5e** exhibited more highly potent antiproliferative activity than the corresponding 2-methylthiazole analogues **3a-e** previously described. These marked differences were maintained in all the biological evaluations performed. In particular, it is important to underline that **4i** has very low toxicity in non tumoral cell lines such as PBLs and HUVECs. Although preliminary investigations regarding the potential antivascular activity of these new oxazoles derivatives indicated that they are not potent vascular disrupting agents, *in vivo* experiments demonstrated that **4i** had excellent antitumor activity that was evident at lower doses than CA-4P and in the absence of obvious toxicity. In summary, the biological characterization of compound **4i** provides compelling evidence to support its further development as an anticancer drug.

## Experimental Section

### Chemistry. Materials and Methods

^1^H and ^13^C NMR data were obtained with a Varian VXR 200 spectrometer and a Varian Mercury Plus 400 spectrometer, respectively. Peak positions are given in parts per million (*δ*) downfield, and *J* values are given in hertz. Positive-ion electrospray ionization (ESI) mass spectra were recorded on a double-focusing Finnigan MAT 95 instrument with BE geometry. Melting points (mp) were determined on a Buchi-Tottoli apparatus and are uncorrected. The purity of tested compounds was determined by combustion elemental analyses conducted by the Microanalytical Laboratory of the Chemistry Department of the University of Ferrara with a Yanagimoto MT-5 CHN recorder elemental analyzer. All tested compounds yielded data consistent with a purity of at least 95% as compared with the theoretical values. TLC was carried out using glass plates coated with silica gel 60 F_254_ by Merck, and compounds were visualized by UV detection or with aqueous KMnO_4_. Flash column chromatography was performed using 230–400 mesh silica gel and the indicated solvent system. Organic solutions were dried over anhydrous Na_2_SO_4_. Solvents and reagents that are commercially available were purchased from Aldrich (Sigma-Aldrich) or Alfa Aesar (Johnson Matthey Company) and were used without further purification unless otherwise noted.

### General procedure A for the preparation of compounds 8 and 9a-f

A mixture of the appropriate 2-bromoacetophenone **6** or **7a-f** (4 mmol) and acetamide (708 mg, 12 mmol) was heated to 150 °C for 2 h. After this time, the mixture was cooled to room temperature, treated with a 2 M aqueous solution of Na_2_CO_3_ (10 mL), and the suspension was carefully adjusted to pH 12 with Na_2_CO_3_. The mixture was extracted with EtOAc (2 × 20 mL), the combined organic phase was washed with water (10 mL) and brine (10 mL), dried (Na_2_SO_4_) and concentrated under reduced pressure. The residue was purified by flash column chromatography on silica gel.

### 4-(3,4,5-Trimethoxyphenyl)-2-methyloxazole (8)

Following general procedure A, the crude residue purified by flash chromatography, using EtOAc:petroleum ether 1:1 (v:v) for elution, yielded **8** as a white solid. Yield 72%, mp 98–100 °C. ^1^H-NMR (CDCl_3_) δ: 2.57 (s, 3H), 3.87 (s, 3H), 3.93 (s, 6H), 6.96 (s, 2H), 7.79 (s, 1H). MS (ESI): [M + 1]^+^ = 250.3.

### 2-Methyl-4-(naphthalen-3-yl)oxazole (9a)

Following general procedure A, the crude residue purified by flash chromatography, using EtOAc:petroleum ether 1:9 (v:v) for elution, yielded **9a** as an orange solid. Yield 71%, mp 72–73 °C. ^1^H-NMR (CDCl_3_) δ: 2.58 (s, 3H), 7.49 (m, 2H), 7.74 (dd, *J* = 8.6 and 1.8 Hz, 1H), 7.93 (m, 4H), 8.28 (s, 1H). MS (ESI): [M + 1]^+^ = 210.3.

### 4-(4-Fluorophenyl)-2-methyloxazole (9b)

Following general procedure A, the crude residue purified by flash chromatography, using EtOAc:petroleum ether 3:7 (v:v) for elution, yielded **9b** as an orange solid. Yield 61%, mp 54–56 °C. ^1^H-NMR (CDCl_3_) δ: 2.53 (s, 3H), 7.08 (t, *J* = 8.8 Hz, 2H), 7.65 (dd, *J* = 8.8 Hz, 2H), 7.76 (s, 1H). MS (ESI): [M + 1]^+^ = 178.2.

### 4-(4-Chlorophenyl)-2-methyloxazole (9c)

Following general procedure A, the crude residue purified by flash chromatography, using EtOAc:petroleum ether 2:8 (v:v) for elution, yielded **9c** as a white solid. Yield 62%, mp 88–90 °C. ^1^H-NMR (CDCl_3_) δ: 2.54 (s, 3H), 7.36 (d, *J* = 8.8 Hz, 2H), 7.64 (d, *J* = 8.8 Hz, 2H), 7.81 (s, 1H). MS (ESI): [M + 1]^+^ = 194.7.

### 2-Methyl-4-*p*-tolyloxazole (9d)

Following general procedure A, the crude residue purified by flash chromatography, using EtOAc:petroleum ether 3:7 (v:v) for elution, yielded **9d** as an orange solid. Yield 63%, mp 49–51 °C. ^1^H-NMR (CDCl_3_) δ: 2.36 (s, 3H), 2.53 (s, 3H), 7.18 (d, *J* = 8.2 Hz, 2H), 7.58 (d, *J* = 8.2 Hz, 2H), 7.77 (s, 1H). MS (ESI): [M + 1]^+^ = 174.2.

### 4-(4-Methoxyphenyl)-2-methyloxazole (9e)

Following general procedure A, the crude residue purified by flash chromatography, using EtOAc:petroleum ether 4:6 (v:v) for elution, yielded **9e** as a white solid. Yield 67%, mp 68–70 °C. ^1^H-NMR (CDCl_3_) δ: 2.66 (s, 3H), 3.84 (s, 3H), 6.94 (d, *J* = 8.8 Hz, 2H), 7.68 (d, *J* = 8.8 Hz, 2H), 7.77 (s, 1H). MS (ESI): [M + 1]^+^ = 190.1.

### 4-(4-Ethoxyphenyl)-2-methyloxazole (9f)

Following general procedure A, the crude residue purified by flash chromatography, using EtOAc:petroleum ether 3:7 (v:v) for elution, yielded **9 f** as a cream-colored solid. Yield 62%, mp 76–79 °C. ^1^H-NMR (CDCl_3_) δ: 1.42 (t, *J* = 6.8 Hz, 3H), 2.56 (s, 3H), 4.01 (q, *J* = 6.8 Hz, 2H), 6.90 (d, *J* = 8.8 Hz, 2H), 7.62 (d, *J* = 8.8 Hz, 2H), 7.73 (s, 1H). MS (ESI): [M + 1]^+^ = 204.3.

### General procedure B for the preparation of compounds 10 and 11a-f

A solution of the appropriate 2-methyl-4-aryloxazole **8** or **9a-f** (4 mmol) in anhydrous CHCl_3_ (20 mL) was cooled to 0 °C, then *N*-bromosuccinimide (783 mg, 4.4 mmol) was added in small portions. The reaction mixture was allowed to warm slowly to room temperature. After 2 h, the resulting mixture was diluted with CH_2_Cl_2_ (20 mL), washed with a saturated solution of NaHCO_3_ (10 mL), brine (10 mL), dried (MgSO_4_) and evaporated. The residue was purified by column chromatography on silica gel.

### 5-Bromo-4-(3,4,5-trimethoxyphenyl)-2-methyloxazole (10)

Following general procedure B, the crude residue purified by flash chromatography, using EtOAc:petroleum ether 4:6 (v:v) for elution, furnished **10** as a white solid. Yield 73%, mp 108–110 °C. ^1^H-NMR (CDCl_3_) δ: 2.54 (s, 3H), 3.88 (s, 3H), 3.93 (s, 6H), 7.20 (s, 2H). MS (ESI): [M]^+^ = 327.4, [M + 2]^+^ = 329.5.

### 5-Bromo-2-methyl-4-(naphthalen-3-yl)oxazole (11a)

Following general procedure B, the crude residue purified by flash chromatography, using ethyl acetate:petroleum ether 2:8 (v:v) for elution, yielded **11a** as a pink solid. Yield 67%, mp 89–91 °C. ^1^H-NMR (CDCl_3_) δ: 2.56 (s, 3H), 7.49 (m, 2H), 7.82 (m, 4H), 8.05 (dd, *J* = 8.8 and 1.6 Hz, 1H). MS (ESI): [M]^+^ = 268.2, [M + 2]^+^ = 270.1.

### 5-Bromo-4-(4-fluorophenyl)-2-methyloxazole (11b)

Following general procedure B, the crude residue purified by flash chromatography, using EtOAc:petroleum ether 2:8 (v:v) for elution, furnished **11b** as an orange oil. Yield 58%. ^1^H-NMR (CDCl_3_) δ: 2.51 (s, 3H), 7.07 (d, *J* = 8.8 Hz, 2H), 7.87 (dd, *J* = 9.2 and 9.0 Hz, 2H). MS (ESI): [M]^+^ = 256.1, [M + 2]^+^ = 258.1.

### 5-Bromo-4-(4-chlorophenyl)-2-methyloxazole (11c)

Following general procedure B, the crude residue purified by flash chromatography, using EtOAc:petroleum ether 4:6 (v:v) for elution, furnished **11c** as an orange solid. Yield 71%, mp 61–62 °C. ^1^H-NMR (CDCl_3_) δ: 2.52 (s, 3H), 7.38 (d, *J* = 8.4 Hz, 2H), 7.87 (d, *J* = 8.4 Hz, 2H). MS (ESI): [M]^+^ = 272.6, [M + 2]^+^ = 274.6.

### 5-Bromo-2-methyl-4-*p*-tolyloxazole (11d)

Following general procedure B, the crude residue purified by flash chromatography, using ethyl acetate:petroleum ether 3:7 (v:v) for elution, yielded **11d** as white solid. Yield 58%, mp 72–74 °C. ^1^H-NMR (CDCl_3_) δ: 2.38 (s, 3H), 2.53 (s, 3H), 7.21 (d, *J* = 8.2 Hz, 2H), 7.84 (d, *J* = 8.2 Hz, 2H), 7.77 (s, 1H). MS (ESI): [M]^+^ = 252.2, [M + 2]^+^ = 254.2.

### n5-Bromo-4-(4-methoxyphenyl)-2-methyloxazole (11e)

Following general procedure B, the crude residue purified by flash chromatography, using EtOAc:petroleum ether 2:8 (v:v) for elution, furnished **11e** as a white solid. Yield 67%, mp 78–80 °C. ^1^H-NMR (CDCl_3_) δ: 2.54 (s, 3H), 3.85 (s, 3H), 6.94 (d, *J* = 9.2 Hz, 2H), 7.85 (d, *J* = 9.2 Hz, 2H). MS (ESI): [M]^+^ = 266.1, [M + 2]^+^ = 268.0.

### 5-Bromo-4-(4-ethoxyphenyl)-2-methyloxazole (11f)

Following general procedure B, the crude residue purified by flash chromatography, using EtOAc:petroleum ether 3:7 (v:v) for elution, yielded **11f** as a pink solid. Yield 59%, mp 64–66 °C. ^1^H-NMR (CDCl_3_) δ: 1.44 (t, *J* = 7.2 Hz, 3H), 2.52 (s, 3H), 4.01 (q, *J* = 7.2 Hz, 2H), 6.92 (dd, *J* = 6.8 and 2.2 Hz, 2H), 7.62 (dd, *J* = 6.8 and 2.2 Hz, 2H), 7.73 (s, 1H). MS (ESI): [M]^+^ = 282.2, [M + 2]^+^ = 284.2.

### General procedure C for the synthesis of compounds 4a-j and 5a-f

A stirred suspension of 5-bromo-2-methyl-4-aryloxazole **10** or **11a-f** (0.5 mmol) and the appropriate arylboronic acid (0.75 mmol) in dioxane (6 mL containing 2 drops of water) was degassed under a stream of N_2_ over 10 min, then treated with PdCl_2_(DPPF) (41 mg, 0.05 mmol) and CsF (190 mg, 1.25 mmol). The reaction mixture was heated under N_2_ at 45 °C for 30 min, then at 65 °C for 6 h. The reaction mixture was cooled to ambient temperature, diluted with CH_2_Cl_2_ (10 mL), filtered through a pad of celite and evaporated *in vacuo*. The residue was dissolved with CH_2_Cl_2_ (15 mL), and the resultant solution was washed sequentially with water (5 mL) and brine (5 mL). The organic layer was dried and evaporated, and the residue was purified by column chromatography on silica gel.

### 4-(3,4,5-Trimethoxyphenyl)-2-methyl-5-(naphthalen-3-yl)oxazole (4a)

Following general procedure C, the residue was purified by flash chromatography on silica gel using light petroleum ether:EtOAc 1:1 as eluent, affording compound **4a** as a yellow solid. Yield: 58%, mp 104–116 °C. ^1^H-NMR (CDCl_3_) δ: 2.66 (s, 3H), 3.76 (s, 6H), 3.83 (s, 3H), 6.96 (s, 2H), 7.51 (m, 2H), 7.68 (dd, *J* = 8.8 and 1.2 Hz, 1H), 7.84 (m, 3H), 8.1 (d, *J* = 1.2 Hz, 1H). ^13^C NMR (100 MHz, CDCl_3_) δ: 13.97, 56.16 (2C), 60.98, 105.02 (2C), 109.98, 120.17, 121.16, 122.02, 124.12, 125.94, 126.80, 126.86, 127.81, 128.20, 133.13, 137.08, 139.75, 145.48, 153.38 (2C), 160.79. MS (ESI): [M]^+^ = 375.5. Anal. (C_23_H_21_NO_4_) C, H, N.

### 5-(3,4,5-Trimethoxyphenyl)-4-(4-fluorophenyl)-2-methyloxazole (4b)

Following general procedure C, the residue was purified by flash chromatography on silica gel using light petroleum ether:EtOAc 1:1 as eluent, affording compound **4b** as a white solid. Yield: 62%, mp 142–144 °C. ^1^H-NMR (CDCl_3_) δ: 2.60 (s, 3H), 3.78 (s, 6H), 3.83 (s, 3H), 6.85 (s, 2H), 7.06 (t, *J* = 8.8 Hz, 2H), 7.60 (dd, *J* = 8.8 and 8.6 Hz, 2H). ^13^C NMR (100 MHz, CDCl_3_) δ: 14.02, 56.18 (2C), 61.03, 104.87 (2C), 115.72, 115.93, 125.14, 127.29, 128.78, 128.86, 134.56, 138.08, 144.40, 153.44 (2C), 160.36 and 163.98 (*J* = 144.8 Hz), 161.49. MS (ESI): [M]^+^ = 343.4. Anal. (C_19_H_18_FNO_4_) C, H, N.

### 5-(3,4,5-Trimethoxyphenyl)-4-(4-chlorophenyl)-2-methyloxazole (4c)

Following general procedure C, the residue was purified by flash chromatography on silica gel using light petroleum ether:EtOAc 6:4 as eluent, affording compound **4c** as a white solid. Yield: 64%, mp 158–160 °C. ^1^H-NMR (CDCl_3_) δ: 2.66 (s, 3H), 3.80 (s, 6H), 3.84 (s, 3H), 6.87 (s, 2H), 7.34 (d, *J* = 8.4 Hz, 2H), 7.56 (d, *J* = 8.8 Hz, 2H). ^13^C NMR (100 MHz, CDCl_3_) δ: 14.04, 56.21 (2C), 61.04, 105.03 (2C), 126.92, 127.49, 127.82 (2C), 128.90 (2C), 134.32, 135.46, 138.18, 144.14, 153.47 (2C), 160.47. MS (ESI): [M]^+^ = 359.6. Anal. (C_19_H_18_ClNO_4_) C, H, N.

### 4-(3,4,5-Trimethoxyphenyl)-2-methyl-5-*p*-tolyloxazole (4d)

Following general procedure C, the residue was purified by flash chromatography on silica gel using light petroleum ether:EtOAc 6:4 as eluent, affording compound **4d** as a white solid. Yield: 51%, mp 110–112 °C. ^1^H-NMR (CDCl_3_) δ: 2.38 (s, 3H), 2.64 (s, 3H), 3.79 (s, 6H), 3.83 (s, 3H), 6.91 (s, 2H), 7.18 (d, *J* = 8.2 Hz, 2H), 7.50 (d, *J* = 8.2 Hz, 2H). ^13^C NMR (100 MHz, CDCl_3_) δ: 14.02, 21.45, 56.16 (2C), 61.01, 104.90 (2C), 126.18, 126.72 (2C), 127.89, 129.32 (2C), 134.29, 137.85, 138.66, 145.44, 153.32 (2C), 159.99. MS (ESI): [M]^+^ = 339.7. Anal. (C_20_H_21_NO_4_) C, H, N.

### 4-(3,4,5-Trimethoxyphenyl)-5-(4-methoxyphenyl)-2-methyloxazole (4e)

Following general procedure C, the residue was purified by flash chromatography on silica gel using light petroleum ether:EtOAc 1:1 as eluent, affording compound **4a** as a white solid. Yield: 59%, mp 134–136 °C. ^1^H-NMR (CDCl_3_) δ: 2.63 (s, 3H), 3.79 (s, 6H), 3.80 (s, 3H), 3.84 (s, 3H), 6.90 (s, 2H), 6.91 (d, *J* = 8.8 Hz, 2H), 7.54 (d, *J* = 8.8 Hz, 2H). ^13^C NMR (100 MHz, CDCl_3_) δ: 13.96, 55.33, 56.06 (2C), 60.93, 104.68 (2C), 113.98 (2C), 121.52, 127.86, 128.36 (2C), 133.57, 137.68, 145.21, 153.25 (2C), 159.70, 160.78. MS (ESI): [M]^+^ = 355.6. Anal. (C_20_H_21_NO_5_) C, H, N.

### 5-(3,4,5-Trimethoxyphenyl)-4-(3-methoxyphenyl)-2-methyloxazole (4f)

Following general procedure C, the residue was purified by flash chromatography on silica gel using light petroleum ether:EtOAc 1:1 as eluent, affording compound **4f** as a white solid. Yield: 64%, mp 104–106 °C. ^1^H-NMR (CDCl_3_) δ: 2.61 (s, 3H), 3.78 (s, 6H), 3.79 (s, 3H), 3.87 (s, 3H), 6.87 (td, *J* = 8.4, 2.8 and 1.2 Hz, 1H), 6.91 (s, 2H), 7.16 (dd, *J* = 2.8 and 1.2 Hz, 1H), 7.21 (dt, *J* = 8.0 and 1.2 Hz, 1H), 7.27 (dd, *J* = 8.4 and 8.0 Hz, 1H). ^13^C NMR (100 MHz, CDCl_3_) δ: 14.12, 55.42, 56.26 (2C), 60.47, 61.02, 105.16 (2C), 112.06, 114.78, 119.27, 126.71, 129.79, 134.35, 138.19, 145.28, 153.38 (2C), 159.77, 160.61. MS (ESI): [M]^+^ = 355.6. Anal. (C_20_H_21_NO_5_) C, H, N.

### 5-(3-Fluoro-4-methoxyphenyl)-4-(3,4,5-trimethoxyphenyl)-2-methyloxazole (4g)

Following general procedure C, the residue was purified by flash chromatography on silica gel using light petroleum ether:EtOAc 1:1 as eluent, affording compound **4g** as a white solid. Yield: 69%, mp 127–129 °C. ^1^H-NMR (CDCl_3_) δ: 2.66 (s, 3H), 3.80 (s, 6H), 3.84 (s, 3H), 3.88 (s, 3H), 6.89 (s, 2H), 6.96 (d, *J* = 8.2 Hz, 1H), 7.36 (m, 2H). ^13^C NMR (100 MHz, CDCl_3_) δ: 14.11, 55.26 (2CC), 56.33, 61.04, 105.01 (2C), 113.29, 114.47, 121.77, 123.02, 127.04, 134.10, 138.15, 144.14, 147.93, 150.96, 153.44 (2C), 160.28. MS (ESI): [M]^+^ = 373.5. Anal. (C_20_H_20_FNO_5_) C, H, N.

### 5-(3-Chloro-4-methoxyphenyl)-4-(3,4,5-trimethoxyphenyl)-2-methyloxazole (4h)

Following general procedure C, the residue was purified by flash chromatography on silica gel using light petroleum ether:EtOAc 6:4 as eluent, affording compound **4h** as a white solid. Yield: 59%, mp 150–152 °C. ^1^H-NMR (CDCl_3_) δ: 2.64 (s, 3H), 3.81 (s, 6H), 3.85 (s, 3H), 3.88 (s, 3H), 6.91 (s, 2H), 6.92 (d, *J* = 8.8 Hz, 1H), 7.49 (dd, *J* = 8.8 and 2.0 Hz, 1H), 7.07 (d, *J* = 2.0 Hz, 1H). ^13^C NMR (100 MHz, CDCl_3_) δ: 13.90, 56.11 (2C), 56.21, 60.94, 104.72 (2C), 111.83, 122.28, 122.65, 126.29, 127.23, 128.41, 134.23, 137.97, 143.76, 153.33 (2C), 155.03, 160.09. MS (ESI): [M]^+^ = 389.4. Anal. (C_20_H_20_ClNO_5_) C, H, N.

### 4-(3,4,5-Trimethoxyphenyl)-5-(4-ethoxyphenyl)-2-methyloxazole (4i)

Following general procedure C, the residue was purified by flash chromatography on silica gel using light petroleum ether:EtOAc 6:4 as eluent, affording compound **4i** as a white solid. Yield: 65%, mp 95–97 °C. ^1^H-NMR (CDCl_3_) δ: 1.43 (t, *J* = 7.0 Hz, 3H), 2.64 (s, 3H), 3.79 (s, 6H), 3.83 (s, 3H), 4.05 (q, *J* = 7.0 Hz, 2H), 6.89 (d, *J* = 8.8 Hz, 2H), 6.92 (s, 2H), 7.53 (d, *J* = 8.8 Hz, 2H). ^13^C NMR (100 MHz, CDCl_3_) δ: 14.03, 14.83, 56.14 (2C), 61.01, 63.62, 104.73 (2C), 114.57 (2C), 122.44, 128.01, 128.43 (2C), 133.61, 137.72, 145.35, 153.32 (2C), 159.25, 159.72. MS (ESI): [M]^+^ = 369.7. Anal. (C_21_H_23_NO_5_) C, H, N.

### 5-(3-Chloro-4-ethoxyphenyl)-4-(3,4,5-trimethoxyphenyl)-2-methyloxazole (4j)

Following general procedure C, the residue was purified by flash chromatography on silica gel using light petroleum ether:EtOAc 6:4 as eluent, affording compound **4j** as a cream-colored solid. Yield: 63%, mp 124–126 °C. ^1^H-NMR (CDCl_3_) δ: 1.49 (t, *J* = 6.8 Hz, 3H), 2.60 (s, 3H), 3.80 (s, 6H), 3.84 (s, 3H), 4.14 (q, *J* = 6.8 Hz, 2H), 6.90 (m, 3H), 7.44 (dd, *J* = 8.4 and 2.4 Hz, 1H), 7.70 (d, *J* = 2.4 Hz, 1H). ^13^C NMR (100 MHz, CDCl_3_) δ: 13.93, 14.70, 56.31 (2C), 61.05, 64.95, 104.91 (2C), 113.03, 121.56, 123.16, 126.50, 128.64, 130.04, 133.34, 138.84, 144.27, 153.51 (2C), 154.93, 160.66. MS (ESI): [M]^+^ = 403.5. Anal. (C_21_H_22_ClNO_5_) C, H, N.

### 5-(3,4,5-Trimethoxyphenyl)-2-methyl-4-(naphthalen-3-yl)oxazole (5a)

Following general procedure C, the residue was purified by flash chromatography on silica gel using light petroleum ether:EtOAc 6:4 as eluent, affording compound **5a** as a white solid. Yield: 63%, mp 95–97 °C. ^1^H-NMR (CDCl_3_) δ: 2.60 (s, 3H), 3.72 (s, 6H), 3.89 (s, 3H), 6.86 (s, 2H), 7.49 (m, 2H), 7.83 (m, 4H), 8.28 (s, 1H). ^13^C NMR (100 MHz, CDCl_3_) δ: 15.46, 56.38 (2C), 61.08, 104.16, 104.67, 124.18, 126.02, 126.48, 127.52, 127.79, 128.08, 128.34, 129.41, 133.13, 133.50, 134.54, 137.66, 138.51, 145.86, 153.45 (2C), 160.44. MS (ESI): [M]^+^ = 375.6. Anal. (C_23_H_21_NO_4_) C, H, N.

### 4-(4-Fluorophenyl)-5-(3,4,5-trimethoxyphenyl)-2-methyloxazole (5b)

Following general procedure C, the residue was purified by flash chromatography on silica gel using light petroleum ether:EtOAc 1:1 as eluent, affording compound **5b** as a white solid. Yield: 63%, mp 132–133 °C. ^1^H-NMR (CDCl_3_) δ: 2.66 (s, 3H), 3.72 (s, 6H), 3.88 (s, 3H), 6.76 (s, 2H), 7.05 (t, *J* = 8.6Hz, 2H), 7.65 (dd, *J* = 8.8 and 8.6 Hz, 2H). ^13^C NMR (100 MHz, CDCl_3_) δ: 14.21, 56.20 (2C), 61.05, 103.68 (2C), 115.47 (2C), 124.10, 128.46, 130.06 (2C), 133.76, 138.37, 145.22, 153.46 (2C), 160.19, 163.86. MS (ESI): [M]^+^ = 343.7. Anal. (C_19_H_18_FNO_4_) C, H, N.

### 4-(4-Chlorophenyl)-5-(3,4,5-trimethoxyphenyl)-2-methyloxazole (5c)

Following general procedure C, the residue was purified by flash chromatography on silica gel using light petroleum ether:EtOAc 7:3 as eluent, affording compound **5c** as a white solid. Yield: 58%, mp 149–151 °C. ^1^H-NMR (CDCl_3_) δ: 2.61 (s, 3H), 3.77 (s, 6H), 3.89 (s, 3H), 6.77 (s, 2H), 7.33 (d, *J* = 8.6 Hz, 2H), 7.62 (d, *J* = 8.8 Hz, 2H). ^13^C NMR (100 MHz, CDCl_3_) δ: 13.75, 56.29 (2C), 61.04, 104.00 (2C), 123.86, 128.78 (2C), 129.41 (2C), 130.59, 133.34, 134.07, 138.61, 145.67, 153.50 (2C), 160.35. MS (ESI): [M]^+^ = 359.6. Anal. (C_19_H_18_ClNO_4_) C, H, N.

### 5-(3,4,5-Trimethoxyphenyl)-2-methyl-4-*p*-tolyloxazole (5d)

Following general procedure C, the residue was purified by flash chromatography on silica gel using light petroleum ether:EtOAc 6:4 as eluent, affording compound **5d** as a white solid. Yield: 55%, mp 128–129 °C. ^1^H-NMR (CDCl_3_) δ: 2.37 (s, 3H), 2.62 (s, 3H), 3.76 (s, 6H), 3.88 (s, 3H), 6.81 (s, 2H), 7.17 (d, *J* = 8.2 Hz, 2H), 7.56 (d, *J* = 8.2 Hz, 2H). ^13^C NMR (100 MHz, CDCl_3_) δ: 14.08, 21.41, 56.16, 56.38, 61.05, 103.65 (2C), 104.68, 124.52, 127.98, 129.20, 129.44, 134.80, 137.67, 138.05, 138.16, 144.88, 153.37, 153.46, 159.95. MS (ESI): [M]^+^ = 339.7. Anal. (C_20_H_21_NO_4_) C, H, N.

### 5-(3,4,5-Trimethoxyphenyl)-4-(4-methoxyphenyl)-2-methyloxazole (5e)

Following general procedure C, the residue was purified by flash chromatography on silica gel using light petroleum ether:EtOAc 7:3 as eluent, affording compound **5e** as a white solid. Yield: 62%, mp 108–110 °C. ^1^H-NMR (CDCl_3_) δ: 2.64 (s, 3H), 3.77 (s, 6H), 3.78 (s, 3H), 3.83 (s, 3H), 6.80 (s, 2H), 6.90 (d, *J* = 8.8 Hz, 2H), 7.61 (d, *J* = 8.8 Hz, 2H). ^13^C NMR (100 MHz, CDCl_3_) δ: 14.18, 55.35, 56.16 (2C), 61.00, 103.69 (2C), 114.22 (2C), 122.84, 128.45, 129.63 (2C), 132.01, 139.21, 145.21, 153.49 (2C), 160.24, 161.11. MS (ESI): [M]^+^ = 355.5. Anal. (C_20_H_21_NO_5_) C, H, N.

### 4-(4-Ethoxyphenyl)-5-(3,4,5-trimethoxyphenyl)-2-methyloxazole (5f)

Following general procedure C, the residue was purified by flash chromatography on silica gel using light petroleum ether:EtOAc 6:4 as eluent, affording compound **5f** as a white solid. Yield: 68%, mp 116–118 °C. ^1^H-NMR (CDCl_3_) δ: 1.39 (t, *J* = 6.8 Hz, 3H), 2.63 (s, 3H), 3.76 (s, 6H), 3.83 (s, 3H), 4.04 (q, *J* = 6.8 Hz, 2H), 6.81 (s, 2H), 6.89 (d, *J* = 8.8 Hz, 2H), 7.59 (d, *J* = 8.8 Hz, 2H). ^13^C NMR (100 MHz, CDCl_3_) δ: 12.34, 15.00, 56.37 (2C), 61.19, 63.74, 103.74 (2C), 104.84, 114.72 (2C), 124.38, 129.69 (2C), 144.78, 147.23, 153.56 (2C), 160.13, 160.98, 171.03. MS (ESI): [M]^+^ = 369.5. Anal. (C_21_H_23_NO_5_) C, H, N.

### Molecular modeling

All molecular modeling studies were performed on a MacPro dual 2.66 GHz Xeon running Ubuntu 14.04. The tubulin structure was downloaded from the PDB data bank (http://www.rcsb.org/; PDB code 1SA0)^38^. Hydrogen atoms were added to the protein, using the Protonate 3D routine of the Molecular Operating Environment (MOE)^39^. Ligand structures were built with MOE and minimized using the MMFF94x force field until a RMSD gradient of 0.05 kcal mol^−1^ Å^−1^ was reached. The docking simulations were performed using PLANTS^40^.

## Biological assays

### Materials and Methods

#### Cell growth conditions and antiproliferative assay

Human T-cell leukemia (Jurkat) and human B-cell leukemia (SEM and RS4;11) cells were grown in RPMI-1640 medium (Gibco, Milano, Italy). Breast adenocarcinoma (MCF-7), human cervix carcinoma (HeLa), human lung adenocarcinoma (A549) and human colon adenocarcinoma (HT-29) cells were grown in DMEM medium (Gibco, Milano, Italy), all supplemented with 115 units/mL penicillin G (Gibco, Milano, Italy), 115 *μ*g/mL streptomycin (Invitrogen, Milano, Italy), and 10% fetal bovine serum (FBS; Invitrogen, Milano, Italy). Stock solutions (10 mM) of the different compounds were obtained by dissolving them in DMSO. Individual wells of a 96-well tissue culture microtiter plate were inoculated with 100 μL of complete medium containing 8 × 10^3^ cells. The plates were incubated at 37 °C in a humidified 5% CO_2_ incubator for 18 h prior to the experiments. After medium removal, 100 μL of fresh medium containing the test compound at different concentrations was added to each well in triplicate and incubated at 37 °C for 72 h. The percentage of DMSO in the medium never exceeded 0.25%. This was also the maximum DMSO concentration in all cell-based assays described below. Cell viability was assayed by the MTT test as previously described.^21^ The IC_50_ was defined as the compound concentration required to inhibit cell proliferation by 50%, in comparison with cells treated with the maximum amount of DMSO (0.25%) and considered as 100% viability.

Peripheral blood lymphocytes (PBL) from healthy donors were obtained from human peripheral blood (leucocyte rich plasma- buffy coats) from healthy volunteers using the Lymphoprep (Fresenius KABI Norge AS) gradient density centrifugation.

Buffy coats were obtained from the Blood Transfusion Service, Azienda Ospedaliera of Padova and provided at this institution for research purposes. Therefore, no informed consent was further needed. In addition, buffy coats were provided without identifiers. The experimental procedures were carried out in strict accordance with approved guidelines.

After extensive washing, cells were resuspended (1.0 × 10^6^ cells/mL) in RPMI-1640 with 10% FBS and incubated overnight. For cytotoxicity evaluations in proliferating PBL cultures, non-adherent cells were resuspended at 5 × 10^5^ cells/mL in growth medium, containing 2.5 *μ*g/mL PHA (Irvine Scientific). Different concentrations of the test compounds were added, and viability was determined 72 h later by the MTT test. For cytotoxicity evaluations in resting PBL cultures, non-adherent cells were resuspended (5 × 10^5^ cells/mL) and treated for 72 h with the test compounds, as described above.

#### Effects on tubulin polymerization and on colchicine binding to tubulin

To evaluate the effect of the compounds on tubulin assembly *in vitro*^41^, varying concentrations of compounds were preincubated with 10 μM bovine brain tubulin in 0.8 M monosodium glutamate (pH adjusted to 6.6 with HCl in a 2.0 M stock solution) at 30 ˚C and then cooled to 0 ˚C. After addition of 0.4 mM GTP, the mixtures were transferred to 0 °C cuvettes in a recording spectrophotometer and warmed to 30 °C. Tubulin assembly was followed turbidimetrically at 350 nm. The IC_50_ was defined as the compound concentration that inhibited the extent of assembly by 50% after a 20 min incubation. The capacity of the test compounds to inhibit colchicine binding to tubulin was measured as described^42^, except that the reaction mixtures contained 1 μM tubulin, 5 μM [^3^H]colchicine and 1 or 5 μM test compound.

#### Flow cytometric analysis of cell cycle distribution

5 × 10^5^ HeLa or Jurkat cells were treated with different concentrations of the test compounds for 24 h. After the incubation period, the cells were collected, centrifuged, and fixed with ice-cold ethanol (70%). The cells were then treated with lysis buffer containing RNase A and 0.1% Triton X-100 and then stained with PI. Samples were analyzed on a Cytomic FC500 flow cytometer (Beckman Coulter). DNA histograms were analyzed using MultiCycle for Windows (Phoenix Flow Systems).

#### Apoptosis assay

Cell death was determined by flow cytometry of cells double stained with annexin V/FITC and PI. The Coulter Cytomics FC500 (Beckman Coulter) was used to measure the surface exposure of PS on apoptotic cells according to the manufacturer’s instructions (Annexin-V Fluos, Roche Diagnostics).

#### Western blot analysis

HeLa cells were incubated in the presence of **4i** and, after different times, were collected, centrifuged, and washed two times with ice cold phosphate buffered saline (PBS). The pellet was then resuspended in lysis buffer. After the cells were lysed on ice for 30 min, lysates were centrifuged at 15000× g at 4 °C for 10 min. The protein concentration in the supernatant was determined using the BCA Protein Assay (Pierce, Italy). Equal amounts of protein (10 μg) were resolved using sodium dodecyl sulfate-polyacrylamide gel electrophoresis (SDS-PAGE) (Criterion Precast, BioRad, Italy) and transferred to a PVDF Hybond-P membrane (GE Healthcare). Membranes were blocked with a bovine serum albumin solution (5% in Tween PBS 1X), the membranes being gently rotated overnight at 4 °C. Membranes were then incubated with primary antibodies against caspase-9, PARP, cdc25c, p-H2AX^Ser139^, cyclin B, p-cdc2^Tyr15^ (all from Cell Signaling) or β-actin (Sigma-Aldrich) for 2 h at room temperature. Membranes were next incubated with peroxidase labeled secondary antibodies for 60 min. All membranes were visualized using ECL Select (GE Healthcare), and images were acquired using an Uvitec-Alliance imaging system (Uvitec, Cambridge, UK). To ensure equal protein loading, each membrane was stripped and reprobed with anti-β-actin antibody.

#### Antivascular activity

HUVECs were prepared from human umbilical cord veins, as previously described^43^. The adherent cells were maintained in M200 medium supplemented with LSGS (Low Serum Growth Supplement), containing FBS, hydrocortisone, hEGF, bFGF, heparin, gentamycin/amphotericin (Life Technologies, Monza, Italy). Once confluent, the cells were detached by a trypsin–EDTA solution and used in experiments from the first to sixth passages.

The motility assay for HUVECs was based on “scratch” wounding of a confluent monolayer^35^. Briefly, HUVECs (1x10^5^) were seeded onto 6-well plates coated with 0.1% collagen type I (BD Biosciences, Italy) in complete medium until a confluent monolayer was formed. The cells were wounded using a pipette tip, and wells were washed with PBS to remove undetached cells. Then, the cells were treated with the test compounds, and, at different times from the scratch, the cells were photographed under a light microscope. At all indicated time points, the wound width was measured in four areas and compared with the initial width.

Matrigel matrix (Basement Membrane Matrix, BD Biosciences, Italy) was kept at 4 °C for 3 h, when 230 μL of Matrigel solution was added to each well of a 24-well plate. After gelling at 37 °C for 30 min, gels were overlaid with 500 μL of medium containing 6 × 10^4^ HUVECs. The cells were incubated over Matrigel for 6 h to allow capillary tubes to form. Different concentrations of test compound were added in the cultures and incubated for different times, and the disappearance of existing vasculature was monitored and photographed (five fields for each well: the four quadrants and the center) at a 10x magnification. Phase contrast images were recorded using a digital camera and saved as TIFF files. Image analysis was carried out using ImageJ image analysis software, and dimensional (percent area covered by HUVECs and total length of HUVECs network per field) and topological parameters (number of meshes and branching points per field) were determined^43^. Values were expressed as percent change from control cultures grown with complete medium.

#### *In vivo* animal studies

Animal experiments were approved by our local animal ethics committee (OPBA, Organismo Preposto al Benessere degli Animali, Università degli Studi di Brescia, Italy) and were executed in accordance with national guidelines and regulations. Procedures involving animals and their care conformed with institutional guidelines that comply with national and international laws and policies (EEC Council Directive 86/609, OJ L 358, 12 December 1987) and with “ARRIVE” guidelines (Animals in Research Reporting *In Vivo* Experiments). Six week old C57BL/6 mice (Charles River, Calco) were injected subcutaneously into the dorsolateral flank with 2.5x10^5^ BL6-B16 murine melanoma cells in 200 μL of PBS. When tumors were palpable, animals were treated intraperitoneally every other day with different doses of test compounds dissolved in 50 μL of DMSO. Tumors were measured in two dimensions, and tumor volume was calculated according to the formula V = (D × d^2^)/2, where D and d are the major and minor perpendicular tumor diameters, respectively.

#### Statistical analysis

Unless indicated differently, the results are presented as mean ± SEM. The differences between different treatments were analyzed, using the two-sided Student’s t test. P values lower than 0.05 were considered significant.

## Additional Information

**How to cite this article**: Romagnoli, R. *et al*. Synthesis and Biological Evaluation of 2-Methyl-4,5-Disubstituted Oxazoles as a Novel Class of Highly Potent Antitubulin Agents. *Sci. Rep.* **7**, 46356; doi: 10.1038/srep46356 (2017).

**Publisher's note:** Springer Nature remains neutral with regard to jurisdictional claims in published maps and institutional affiliations.

## Supplementary Material

Supplementary Information

## Figures and Tables

**Figure 1:**
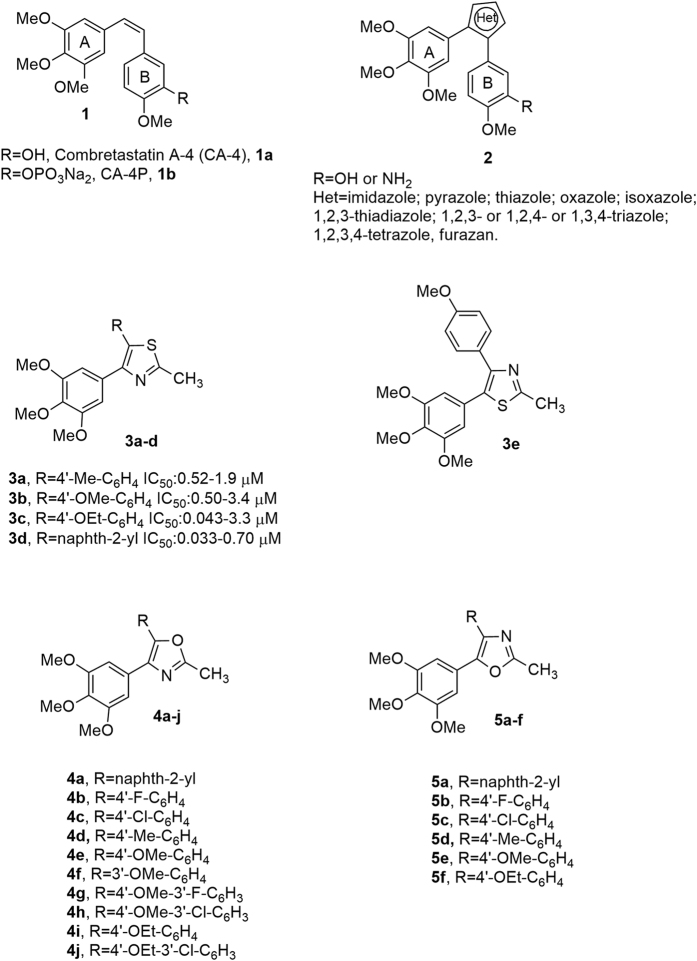
Lead structures of tubulin polymerization inhibitors.

**Table 1:** *In vitro* cell growth inhibitory effects of compounds **3e**, **4a-j**, **5a-f** and CA-4 (**1**).

Compd	IC_50_^a^ (nM)						
	HeLa	A549	HT-29	MCF-7	Jurkat	RS 4;11	SEM
**4a**	2.1 ± 0.5	12.6 ± 5.4	0.5 ± 0.1	1.1 ± 0.5	73.2 ± 5.2	14.3 ± 6.2	40.2 ± 11.0
**4b**	>10000	>10000	>10000	>10000	>10000	>10000	9550 ± 2050
**4c**	2818 ± 169	8785 ± 2309	1397 ± 384	2587 ± 447	5576 ± 888	840 ± 260	2211 ± 256
**4d**	31.0 ± 16.0	92.3 ± 21.5	53.0 ± 24.6	243.8 ± 74.2	102.2 ± 22.6	25.3 ± 3.2	133.8 ± 39.5
**4e**	10.8 ± 4.7	179.3 ± 36.3	40.8 ± 19.7	27.7 ± 12.0	18.7 ± 6.0	7.6 ± 2.3	35.6 ± 12.5
**4f**	> 10000	> 10000	> 10000	> 10000	5233 ± 88.2	5333 ± 166.7	3633 ± 171
**4g**	2.4 ± 0.6	0.65 ± 0.08	3.3 ± 1.1	2.5 ± 0.7	4.6 ± 1.0	0.35 ± 0.15	3.9 ± 0.8
**4h**	168.8 ± 75.5	138.3 ± 13.7	207.0 ± 93.0	143.8 ± 64.3	512.2 ± 92.7	47.0 ± 13.9	233.3 ± 34.6
**4i**	0.9 ± 0.3	0.5 ± 0.1	20.2 ± 8.8	4.5 ± 1.8	4.1 ± 1.1	2.1 ± 0.2	6.1 ± 2.3
**4j**	4.2 ± 1.3	20.3 ± 11.4	0.9 ± 0.5	1.5 ± 0.6	77.7 ± 6.1	20.2 ± 5.4	51.0 ± 13.5
**5a**	37.0 ± 14.4	45.2 ± 12.5	315.2 ± 145.3	5.2 ± 2.4	638.3 ± 153.5	249.5 ± 89.3	37.0 ± 14.4
**5b**	>10000	>10000	>10000	>10000	9000 ± 577.4	>10000	>10000
**5c**	2557 ± 474.6	>10000	>10000	>10000	5250 ± .934	815 ± 405.9	6567 ± 499.6
**5d**	148.2 ± 55.3	2579 ± 289	523.3 ± 61.8	360.0 ± 52.6	921.7 ± 76.6	635.2 ± 387.1	351.7 ± 77.8
**5e**	24.0 ± 10.5	243.3 ± 51.5	12.3 ± 5.9	83.8 ± 20.2	43.6 ± 12.3	17.3 ± 4.2	96.6 ± 22.5
**5f**	2.0 ± 0.8	1.5 ± 0.44	1.9 ± 0.8	0.4 ± 0.08	50.7 ± 10.8	11.9 ± 6.3	13.3 ± 4.2
**3e**	1978 ± 779.7	2758 ± 1203	3700 ± 263	1933 ± 448	7200 ± 586	2972 ± 738.3	1978 ± 779.7
**CA-4**	4 ± 0.1	180 ± 50	3100 ± 100	370 ± 100	5 ± 0.6	0.8 ± 0.2	5 ± 0.1

^a^IC_50_ = compound concentration required to inhibit tumor cell proliferation by 50%. Values are the mean ± SE from the dose-response curves of at least three independent experiments carried out in triplicate.

**Table 2:** Cytotoxicity of **4n** for nontumoral human cells.

	IC_50_ (μM)^a^		
	4a	4i	5f
PBL_resting_^b^	15.5 ± 1.5	14.9 ± 3.5	34.5 ± 4.5
PBL_PHA_^c^	21.1 ± 1.2	18.3 ± 1.5	17.7 ± 2.1
HUVECs	>100	>100	>100

Values are the mean ± SEM for three separate experiments.

^a^Compound concentration required to reduce cell growth by 50%.

^b^PBL not stimulated with PHA.

^c^PBL stimulated with PHA.

**Table 3:** Inhibition of tubulin polymerization and colchicine binding by compounds **4a, 4d-e, 4 g**, **4i-j**, **5a**, **5e-f** and CA-4.

Compound	Tubulin assembly^a^ IC_50_ ± S.D (μM)	Colchicine binding%^b^ ± S.D	
		5 μM drug	1 μM drug
**4a**	1.2 ± 0.1	74 ± 5	47 ± 0.8
**4d**	0.66 ± 0.0	82 ± 3	55 ± 3
**4e**	0.95 ± 0.1	78 ± 3	47 ± 4
**4 g**	1.2 ± 0.0	78 ± 0.5	51 ± 1
**4i**	0.56 ± 0.1	86 ± 1	62 ± 0.4
**4j**	1.8 ± 0.1	53 ± 3	n.d.
**5a**	1.6 ± 0.1	64 ± 4	n.d.
**5e**	2.6 ± 0.3	54 ± 3	n.d.
**5f**	1.3 ± 0.1	72 ± 2	n.d.
CA-4 (**1**)	1.3 ± 0.1	99 ± 0.7	93 ± 0.8

n.d. = not determined.

^a^Inhibition of tubulin polymerisation. Tubulin was at 10 μM.

^b^Inhibition of [^3^H] colchicine binding. Tubulin and colchicine were at 1 and 5 μM concentrations, respectively.
