# Supplementary material for: Synthesis and Biological Evaluation of 2-Methyl-4,5-Disubstituted Oxazoles as a Novel Class of Highly Potent Antitubulin Agents
Source: Sci Rep. 2017 Apr 13;7:46356. doi: 10.1038/srep46356 (PMC5390315; doi:10.1038/srep46356)
Supplement: Supplementary Information [file srep46356-s1.pdf]

Synthesis and Biological Evaluation of 2-Methyl-4,5-Disubstituted Oxazoles as a Novel Class of Highly Potent Antitubulin Agents

Romeo Romagnoli,[1]* Pier Giovanni Baraldi,[1] Filippo Prencipe,[1] Paola Oliva,[1] Stefania Baraldi,[1] Maria Kimatrai Salvador,[2] Luisa Carlota Lopez-Cara,[2*] Andrea Brancale,[3] Salvatore Ferla,[3] Ernest Hamel,[4] Roberto Ronca,[5] Roberta Bortolozzi,[6] Elena Mariotto,[6] Elena Porcù,[6] Giuseppe Basso[6] and Giampietro Viola[6]*

[1]Dipartimento di Scienze Chimiche e Farmaceutiche, Università di Ferrara, 44121 Ferrara, Italy; [2]Departamento de Química Farmacéutica y Orgánica, Facultad de Farmacia, Campus de Cartuja s/n, 18071, Granada, Spain; [3]School of Pharmacy and Pharmaceutical Sciences, Cardiff University, King Edward VII Avenue, Cardiff, CF10 3NB, UK; [4]Screening Technologies Branch, Developmental Therapeutics Program, Division of Cancer Treatment and Diagnosis, Frederick National Laboratory for Cancer Research, National Cancer Institute, National Institutes of Health, Frederick, Maryland 21702, USA; [5]Dipartimento di Medicina Molecolare e Traslazionale Unità di Oncologia Sperimentale ed Immunologia, Università di Brescia, 25123 Brescia, Italy; [6]Dipartimento di Salute della Donna e del Bambino, Laboratorio di Oncoematologia, Università di Padova, 35131 Padova, Italy

SUPPORTING INFORMATION

Synthetic procedure for the preparation of compound **3e**

Raw images Western blots Figures S1-S2

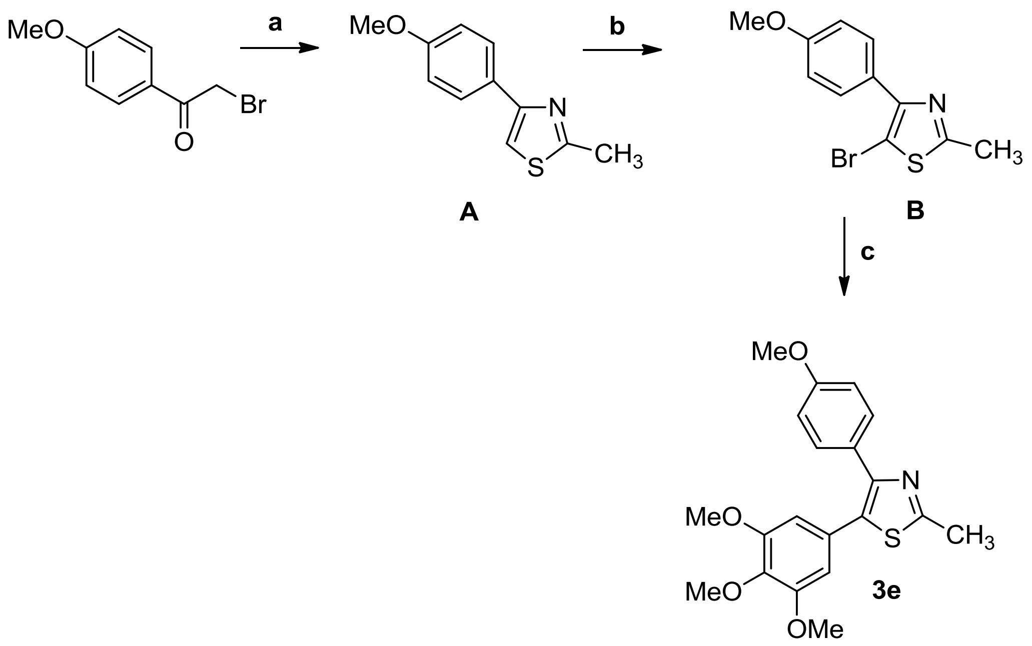

Reagents. a: CH_3CONH_2, 150 °C, 2 h; **b**: NBS, $CHCl_3$, rt; **c**: $PdCl_2$(DPPF), $(OMe)_3C_6H_2B(OH)_2$, CsF, 1,4-dioxane. 65 °C.

4-(4-Methoxyphenyl)-2-methylthiazole (**A**). A mixture of 2-bromo-1-(4-methoxyphenyl)ethanone (916 m, 4 mmol) and thioacetamide (331 mg, 5.5 mmol, 1.1 equiv.) in anhydrous EtOH (15 mL) was heated to reflux for 2 h. After that, the solvent was removed *in vacuo*, and saturated aqueous $NaHCO_3$ (5 mL) was added to make the mixture basic (pH=8-9). Then the mixture was extracted with EtOAc (3 x 10 mL). The combined organic phases were washed with water (10 mL) and brine (10 mL), dried with anhydrous Na_2SO_4 and concentrated under vacuum. The crude residue purified by flash chromatography, using EtOAc:petroleum ether 1:1 (v:v) for elution, yielded compound **A** as a white solid. Yield 72%, mp 68-70 °C. ^{1}H-NMR ($CDCl_3$) δ: 2.77 (s, 3H), 3.84 (s, 3H), 6.91 (d, J=8.8 Hz, 2H), 7.17 (s, 1H), 7.79 (d, J=8.8 Hz, 2H). MS (ESI): $[M+1]^+$=206.3.

5-Bromo-4-(4-methoxyphenyl)-2-methylthiazole (**B**). A solution of 4-(4-methoxyphenyl)-2-methylthiazole **A** (410 mg, 2 mmol) in chloroform (10 mL) was cooled to 0 °C and treated with *N*-bromosuccinimide (392 mg, 2.2 mmol) under nitrogen. The reaction was allowed to warm to room temperature and then stirred for 4 h. After quenching with saturated $Na_2S_2O_3$ (5 mL), the resulting

mixture was diluted with dichloromethane (10 mL). The organic phase was washed with water (5 mL) and brine (5 mL), dried ($MgSO_4$) and evaporated. The crude residue purified by flash chromatography, using EtOAc:petroleum ether 2:8 (v:v) for elution, furnished compound **B** as a pink solid. Yield 73%, mp 64-66 °C. ^{1}H-NMR ($CDCl_3$) δ: 2.69 (s, 3H), 3.85 (s, 3H), 6.94 (d, *J*=8.8 Hz, 2H), 7.83 (d, *J*=8.8 Hz, 2H). MS (ESI): $[M]^+$=283.1, $[M+2]^+$=285.0.

4-(4-Methoxyphenyl)-2-methyl-5-(3,4,5-trimethoxyphenyl)thiazole (3e). A stirred suspension of 5-bromo-4-(4-methoxyphenyl)-2-methylthiazole **B** (142 mg, 0.5 mmol) and (3,4,5-trimethoxyphenyl)boronic acid (212 mg, 1 mmol) in dioxane (6 mL containing 3 drops of water) was degassed under a stream of nitrogen for 10 min, then treated with [1, 1'-bis(diphenylphosphino)ferrocene] dichloropalladium (II) methylene chloride complex (41 mg, 0.05 mmol) and cesium fluoride (190 mg, 1.25 mmol). The reaction mixture was heated under nitrogen at 45 °C for 30 min, then at 65 °C for 5 h. The reaction mixture was cooled to ambient temperature, diluted with CH_2Cl_2 (10 mL), filtered through a pad of celite and evaporated *in vacuo*. The residue was dissolved with CH_2Cl_2 (15 mL), and the resultant solution was washed sequentially with water (5 mL) and brine (5 mL). The organic layer was dried and evaporated, and the residue was purified by flash chromatography on silica gel using light petroleum ether: EtOAc 7:3 as eluent, affording compound **3e** as a white solid. Yield: 65%, 93-95 ºC. ^{1}H-NMR ($CDCl_3$) δ: 2.80 (s, 3H), 3.71 (s, 6H), 3.80 (s, 3H), 3.89 (s, 3H), 6.52 (s, 2H), 6.71 (s, 1H), 6.82 (d, *J*=8.8 Hz, 2H), 7.46 (d, *J*=8.8 Hz, 2H). ^{13}C NMR (100 MHz, $CDCl_3$) δ: 18.88, 55.37, 56.25, 56.36, 61.05, 104.65, 106.80 (2C), 113.92 (2C), 126.69, 129.85, 130.59 (2C), 131.28, 137.64, 138.24, 153.42, 159.72 (2C). MS (ESI): $[M]^+$=371.6. Anal. ($C_{20}H_{21}NO_4S$) C, H, N.

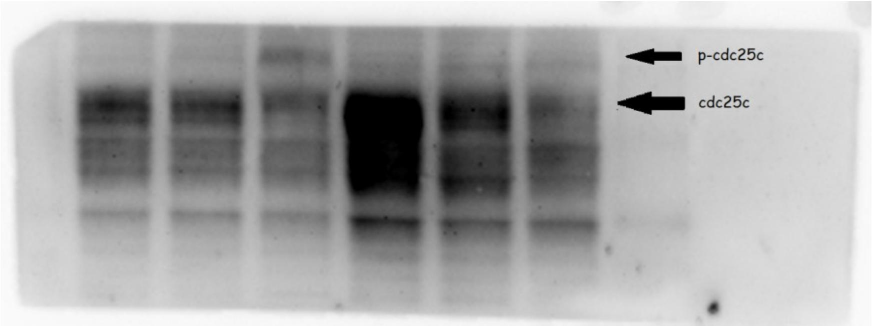

Cdc25C

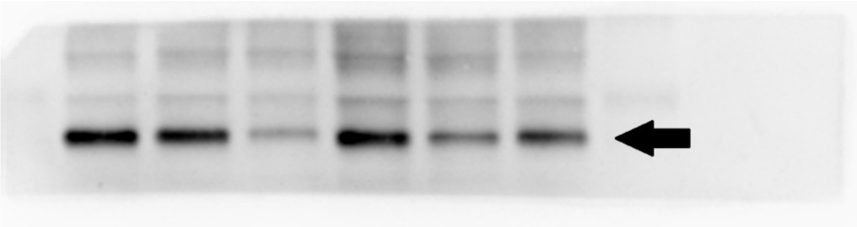

p-Cdc2 (Y15)

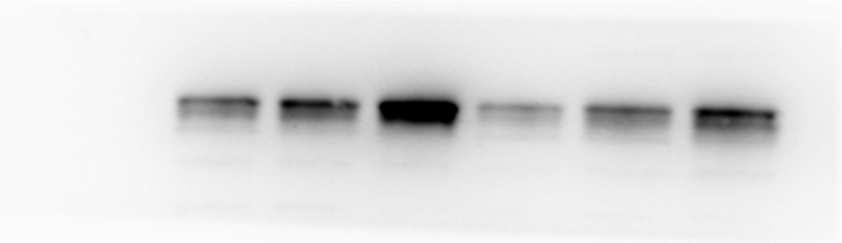

Cyclin B

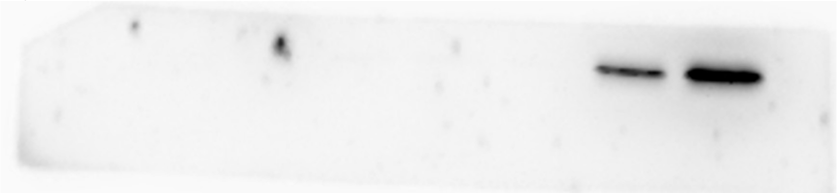

H2AX

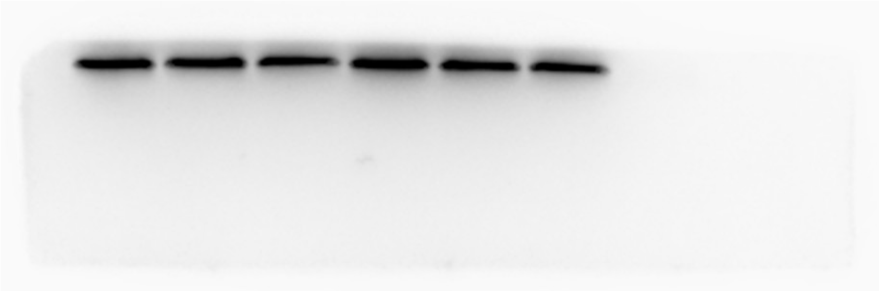

Actin

Figure S1
Raw imges of Western blots in figure 5. All proteins were identified by appropriate molecular marker runned together. Arrows indicate the protein of interest.

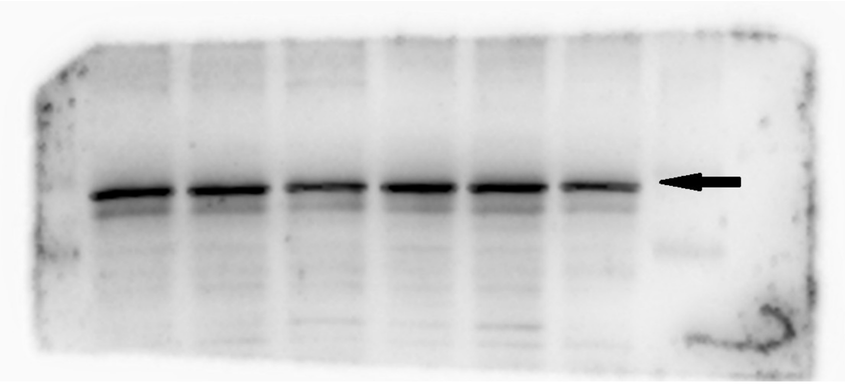

Mcl-1

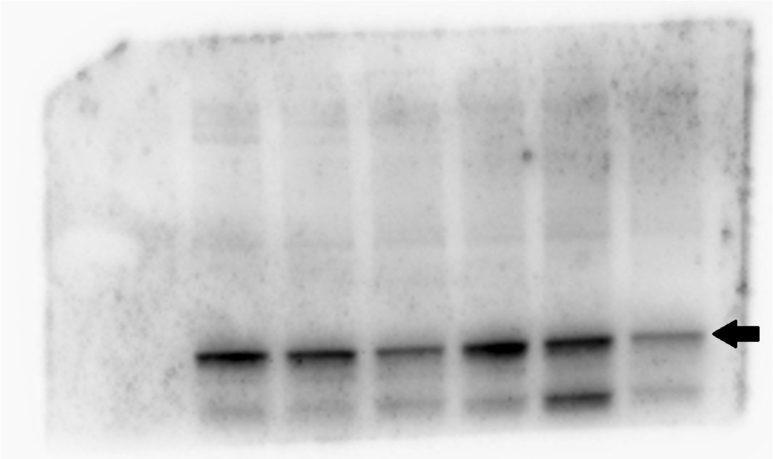

XIAP

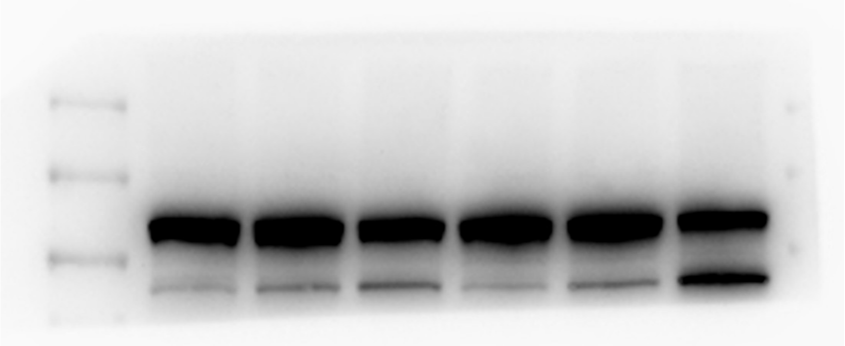

PARP and cleaved PARP

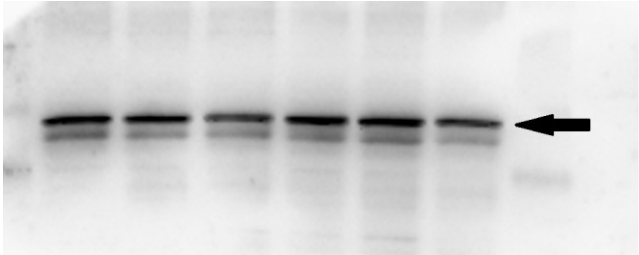

Actin

Figure S2
Raw imges of Western blots in figure 8. All proteins were identified by appropriate molecular marker runned together. Arrows indicate the protein of interest.
